# C-terminal Fragment Generated by HOIL-1 Cleavage Suppresses Inflammatory Responses of Myeloid Cells to Alleviate Colitis

**DOI:** 10.7150/thno.124294

**Published:** 2026-02-11

**Authors:** Xiaomeng Li, Hefan Zhang, Qian Wang, Qianqian Li, Xingru Wang, Yu Tian, Rui Zhang, Qiuyun Chen, Christopher M. Overall, Stuart E. Turvey, Bangmao Wang, Hailong Cao, Hong Yang, Shan-Yu Fung

**Affiliations:** 1 State Key Laboratory of Experimental Hematology, Department of Immunology and Key Laboratory of Immune Microenvironment and Disease (Ministry of Education), School of Basic Medical Science, The Province and Ministry Co-Sponsored Collaborative Innovation Center for Medical Epigenetics, International Joint Laboratory of Ocular Diseases, Ministry of Education, Tianjin Medical University, Tianjin, China.; 2 Department of Biochemistry and Molecular Biology, Department of Oral Biological and Medical Science, Center for Blood Research, The University of British Columbia, Vancouver, Canada.; 3 Department of Pediatrics, British Columbia Children's Hospital, Experimental Medicine Program, Faculty of Medicine, University of British Columbia, Vancouver, Canada.; 4 Department of Gastroenterology and Hepatology, General Hospital, Tianjin Medical University, Tianjin Institute of Digestive Diseases, Tianjin Key Laboratory of Digestive Diseases, Tianjin, China.; 5 Department of Pharmacology and Tianjin Key Laboratory of Inflammatory Biology, School of Basic Medical Sciences, The Province and Ministry Co-Sponsored Collaborative Innovation Center for Medical Epigenetics, International Joint Laboratory of Ocular Diseases, Ministry of Education, Intensive Care Unit of the Second Hospital, Tianjin Medical University, Tianjin, China.

**Keywords:** inflammatory bowel disease, macrophage, HOIL-1 cleavage, STAT1, immunotherapy

## Abstract

**Rationale:**

Deciphering the molecular consequences of protein cleavage in inflammatory signaling is vital for defining the mechanisms of intestinal autoinflammation and identifying new therapeutic targets for inflammatory bowel disease (IBD). While it was previously established that HOIL-1 cleavage by MALT1 negatively regulates NF-κB activation and inflammatory responses *in vitro*, the pathophysiological role of HOIL-1 cleavage in regulating intestinal inflammation and the specific function of the resulting C-terminal fragment (C-HOIL-1) remained elusive. This study aimed to define the role of HOIL-1 cleavage and C-HOIL-1 in modulating gut inflammation.

**Methods:**

To investigate the impact of HOIL-1 cleavage on intestinal inflammation, the global and myeloid-specific transgenic mouse models with uncleavable HOIL-1 (lacking C-HOIL-1) were established, and their disease phenotypes and immune profiles were characterized under DSS-induced colitis. Genetically engineered THP-1 monocytic cells expressing uncleavable HOIL-1 and C-HOIL-1 were constructed to elucidate the molecular mechanisms of C-HOIL-1 in regulating inflammatory signaling. Finally, Lenti-C-HOIL-1 was delivered to the colon of wild-type mice via enema to evaluate the therapeutic potential of C-HOIL-1 in controlling intestinal inflammation.

**Results:**

Mice with uncleavable HOIL-1 (lacking C-HOIL-1) present a more severe disease phenotype in DSS-induced colitis; specifically, the infiltration of inflammatory monocytes, M1-type macrophages, and neutrophils is significantly elevated in the colon. Mechanistically, we discover that C-HOIL-1 has novel biological functions in i) inhibiting NF-κB signaling, ii) interacting with STAT1 to down-regulate STAT1-mediated inflammatory signaling, and iii) up-regulating *ARG1* expression. Collectively, these actions suppress the inflammatory responses in monocytes/macrophages, and impede the differentiation of M1-type macrophages. The pretreatment of Lenti-C-HOIL-1 to the colon of wild-type mice alleviates DSS-induced intestinal inflammation.

**Conclusions:**

Our results define the pathophysiological role of HOIL-1 cleavage in colitis, and unveil new functions of C-HOIL-1 in regulating myeloid inflammatory responses. These findings provide a potential therapeutic strategy for controlling gut inflammation in IBD.

## Introduction

Innate immune signaling crosstalk is intricate but essential for maintaining immune homeostasis. Dysregulated inflammatory responses are a hallmark of many autoinflammatory diseases such as inflammatory bowel disease (IBD) 1-3. Thus, identifying intrinsic regulatory factors within such complex signaling networks is critical for elucidating disease mechanisms and finding new therapeutic targets. The transcription factor nuclear factor κB (NF-κB) pathway is one master regulator in immune responses, and defects in this pathway can lead to many autoinflammatory conditions. For example, Blau syndrome 4 and the recently defined Otulipenia/Otulin-related autoinflammatory syndrome (ORAS) are caused by hyper-activation of NF-κB 5-7. Specifically, ORAS results from a loss of function in the deubiquitinase OTULIN, leading to sustained linear ubiquitination and excessive tumor necrosis factor receptor (TNFR)-mediated NF-κB activation 8. Intriguingly, human deficiency in the linear ubiquitin chain assembly complex (LUBAC) that is responsible for catalyzing linear ubiquitination to assist NF-κB activation also manifests with autoinflammatory episodes 9, 10. This evidence suggests that the regulation of NF-κB pathway requires precise integration of opposing signals to control inflammatory responses. Therefore, identifying key regulators that bridge these signaling pathways is critical to deciphering how immune homeostasis is maintained.

Deficiency in heme-oxidized iron regulatory protein 2 (IRP2) ubiquitin ligase 1 (HOIL-1) is featured with clinical manifestations of autoinflammation 9. As a scaffold protein, HOIL-1 binds to HOIL-1 interacting protein (HOIP) and SHANK-associated RH domain-interacting protein (SHARPIN) to maintain the stability and catalytic activity of LUBAC 11, 12. Patients with HOIL-1 deficiency have impaired LUBAC function and attenuated NF-κB activation in B cells and fibroblasts 9, 10. In contrast, the peripheral mononuclear cells (PBMCs) from these patients exhibit a hyper-response to interleukin 1β (IL-1β), but not to TNF-α 9, which may contribute to the autoinflammatory episodes. These clinical observations suggest that HOIL-1 possesses distinct, undefined functions in myeloid cells, which are essential for the regulation of inflammatory responses.

We and others previously demonstrated that HOIL-1 is cleaved by MALT1, the only human paracaspase, to down-regulate NF-κB activation *in vitro* 13-15. This cleavage occurs at arginine 165 (R165) to generate N-terminal (N-HOIL-1) and C-terminal (C-HOIL-1) fragments. While N-HOIL-1 contains the ubiquitin-like (UBL) domain required for interacting with other LUBAC components, C-HOIL-1 is released from the complex. Although HOIL-1 cleavage is known for destabilizing LUBAC, which consequently reduces LUBAC catalytic activity and abrogates NF-κB activation 13-15, whether the released C-HOIL-1 exerts independent biological functions remains elusive. On the other hand, preventing HOIL-1 cleavage by mutating R165 to lysine (K) (HOIL-1-R/K) can up-regulate LUBAC activity and NF-κB signaling 13, which subsequently elevates inflammatory gene expression in human fibroblasts 16. Despite these findings of the regulatory implications of HOIL-1 cleavage, its pathophysiological role *in vivo* has yet to be defined.

Beyond the role in destabilizing LUBAC, we hypothesized that HOIL-1 cleavage and the resulting C-HOIL-1 fragment have novel biological functions that have never been defined. This hypothesis is prompted by paradoxical clinical findings in immunodeficient patients. It has been found that human MALT1 deficiency presents a unique feature of spontaneous inflammation throughout the digestive track in analogy to the pathology of IBD 17-21; mice with protease-dead MALT1 also develop gastrointestinal inflammation over time (> 10 weeks) 22. These imply that the inability to cleave HOIL-1 (i.e., lack of C-HOIL-1) may lead to serious autoinflammation in the gut. On the other hand, HOIL-1 deficient patients (also lack of C-HOIL-1) present hyper-inflammatory responses in peripheral mononuclear cells 9. The fact that both impaired HOIL-1 cleavage and HOIL-1 deficiency result in intestinal autoinflammation suggests that C-HOIL-1 is indispensable for regulating inflammatory responses of myeloid cells in the gut.

To disclose the uncharacterized role of C-HOIL-1 in regulating intestinal inflammation, we constructed an uncleavable HOIL-1 knock-in mouse model (HOIL-1-R/K or designated as R/K; lack of C-HOIL-1). Under the condition of the dextran sulfate sodium (DSS)-induced experimental colitis, we found that R/K mice presented a more severe disease phenotype in comparison with the wild-type (WT) mice. The multi-color flow cytometry analysis revealed the increased infiltration of inflammatory Ly6C^hi^CX3CR1^low^ monocytes, M1-type macrophages, and neutrophils in the colonic lamina propria of R/K mice. Using the genetically engineered HOIL-1-R/K THP-1 monocytes, we confirmed that the NF-κB signaling pathway was hyper-activated in comparison with the WT-HOIL-1. Next, the lentiviral transduction to restore C-HOIL-1 in the R/K THP-1 monocytes inhibited TNF-α-induced NF-κB activation and the pro-inflammatory cytokine production, including IL-8 and CCL4 (also known as macrophage inflammatory protein-1β or MIP-1β). Mechanistic studies using immunoprecipitation and quantitative mass spectrometry revealed that C-HOIL-1 could directly bind to and inhibit signal transducer and activator of transcription 1 (STAT1), thereby down-regulating the downstream interferon (IFN) stimulated gene (ISG) expression. Moreover, the inhibition of STAT1 signaling by C-HOIL-1 abrogated classical inflammasome activation in macrophages. Interestingly, C-HOIL-1 intrinsically up-regulated Arginase 1 (ARG1) expression. These findings for the first time demonstrated that C-HOIL-1 is biologically functional in regulating the inflammatory responses and the differentiation of M1-type macrophages. Such a specific action in myeloid cells was confirmed in a conditional HOIL-1-R/K knock-in mouse model. Finally, the delivery of Lenti-C-HOIL-1 to the colon of WT mice via enema effectively ameliorated DSS-induced colitis. This work elucidates the essential mechanism of HOIL-1 cleavage and the resulting C-HOIL-1 fragment in regulating myeloid cell inflammatory responsiveness, and suggests a novel molecular strategy for controlling gut inflammation in IBD.

## Materials and Methods

### Materials

The THP-1 cell line was obtained from ATCC (Rockefeller, MD, USA). The cell culture medium (RPMI 1640 and DMEM), phosphate buffered saline (PBS) and fetal bovine serum (FBS) were from Biological Industries (Kibbutz Beit Haemek, Israel). The IMDM medium, sodium pyruvate, and L-glutamine were purchased from Gibco (Grand Island, NY, USA). The dextran sulfate sodium (DSS) (36,000-50,000 MW) was obtained from MP Biomedicals (Santa Ana, CA, USA). Mouse ELISA kits (IL-6, TNF-α, CCL2, and IL-10) and human ELISA kits for CCL4 and IL-8, were all obtained from Invitrogen (Grand Island, NY, USA), whereas mouse ELISA kits for CXCL1/KC and CCL4 were from R&D Systems (Minneapolis, MN, USA). The recombinant human TNF-α and IFN-β were from PeproTech of Thermo Fisher Scientific (Cranbury, NJ, USA), while human IFN-γ and IL-4 were from MCE (Monmouth Junction, NJ, USA). Bovine serum albumin (BSA) was obtained from Genview (Houston, TX, USA). The sodium citrate tribasic dihydrate and Tween 20 were purchased from Sangon Biotech (Shanghai, China). The Coomassie Plus (Pierce) for Bradford assay, Halt protease and phosphatase inhibitor cocktail, and TRIZOL were from Thermo Fisher Scientific (Waltham, MA, USA). Ethylenediaminetetraacetic acid (EDTA), 1× HBSS (Hank's balanced salt solution, excluding Ca²⁺ and Mg²⁺), HEPES, collagenase IV, DNaseI, 2-(4-Amidinophenyl)-6-indolecarbamidine dihydrochloride (DAPI), Percoll, dithiothreitol (DTT), puromycin, and Triton X-100 were all purchased from Solarbio (Beijing, China). Chloroform was purchased from Kermel (Tianjin, China). Paraformaldehyde was from Life Lab Biotech (Shanghai, China) and Hoechst 33258 was obtained from Beyotime (Shanghai, China). PEI transfection reagent, phorbol 12-myristate 13-acetate (PMA), and FLAG magnetic beads were purchased from Sigma Aldrich (Sant-Louis, MO, USA). Protein A/G magnetic beads were from Millipore (LSKMAGAG, Darmstadt, Germany). TUBE2 magnetic beads, PR-619, and 1,10 Phenanthroline were obtained from LifeSensors (Malvern, PA, USA). SYBR qPCR Mix was purchased from GenStar, (Suzhou, China).

### Animal studies

HOIL-1-R165K and HOIL-1-R165K^flox/flox^ (as R/K^flox/flox^) transgenic mice (C57BL/6J) via CRISPR-Cas9 technology were obtained from the GemPharmatech (Suzhou, China). Lyz2-Cre^+^ mice were kindly provided by Professor Qiujing Yu. Transgenic mice were in-house bred from F2 generation in the Tianjin Medical University Animal Laboratory Center (SPF level). R/K^flox/flox^Lyz2-Cre^+^ mice were generated by crossing the Lyz2-Cre^+^ mice with R/K^flox/flox^ mice for myeloid-specific uncleavable HOIL-1. The genotype of each mouse was identified (wild-type, heterozygous or homozygous HOIL-1-R165K) through Sanger sequencing of the *Rbck1* gene (encoding HOIL-1) or through PCR of characteristic bands (for R/K^flox/flox^Lyz2-Cre^+^ mice) with the primer sequences listed in **Table S1**. Wild-type mice for C-HOIL-1 lentiviral particle pretreatment were obtained from Beijing Vital River Laboratory Animal Technology (Beijing, China) and kept in the in the Tianjin Medical University Animal Laboratory Center (SPF level) prior to experiments.

### DSS-induced colitis mouse model

Adult mice (8 to 9 weeks, 18-20 g, female) of different genotypes were housed in cages for at least 1 week before the colitis induction. To induce the experimental colitis, mice were given with 2.5% DSS in drinking water for continuous 7 days, and then fed with normal drinking water for another 2 days. The control mice were given with drinking water for 9 days continuously for comparison. During the 9-day model period, the disease activity index (DAI), including weight loss, stool morphology and rectal bleeding, was assessed daily according to the criteria listed in **Table S2**. On day 9, mice were sacrificed, and the colon was collected for the measurement of the colon length, and the colon tissues were processed for further studies.

To evaluate the therapeutic potential of C-HOIL-1 *in vivo*, WT female mice (8 to 9 weeks, 18-20 g) were fasted for 24 h before the pretreatment of C-HOIL-1 lentiviral particles via enema. Mice were anesthetized with 2,2,2-Tribromoethanol (Macklin, Shanghai, China), and then treated with 50% ethanol (100 µL) by enema to increase mucosal permeability. Four hours later, C-HOIL-1-GFP lentiviral particles, the control empty lentiviral particles (V-GFP) or PBS were given to the mice by the same route. Each mouse was injected with 100 µL solution (PBS) containing lentiviral particles with a titer of 800 ng viral P24 protein. One day after C-HOIL-1 treatment, the DSS-induced colitis model was applied as described above.

### Histological analysis on the colon inflammation and injury

Small fragments of the colon tissue were collected, fixed and dehydrated for paraffin embedding to prepare 4-μm thick tissue sections. These sections were then dewaxed and re-hydrated, followed by hematoxylin and eosin (H&E) staining and imaging under an optical microscope (Olympus, BX51, Tokyo, Japan), where 8 images (200×) from different fields were taken for each section with 3 sections for each sample. Histological scoring was performed by two blinded individuals independently following the criteria listed in **Table S3** on 7 features: loss of goblet cells, sub-mucosal edema, crypt abscesses, extent of crypt damage, infiltration of inflammatory cells, extent of inflammation, and reactive epithelial hyperplasia.

### Immune cell isolation from the colonic lamina propria

At the end of DSS-induced colitis model, colon tissues (about 4 cm) were harvested to collect immune cells from the lamina propria for cell infiltration analysis. The tissues were cut into small pieces and digested in 1× HBSS (Hank's balanced salt solution, excluding Ca²⁺ and Mg²⁺) separation solution containing 5% FBS, 2 mM EDTA, 1 mM DTT, and 10 mM HEPES at 37 ℃ for 30 minutes. After centrifugation, the precipitates were further treated with 1x HBSS digestion solution (containing 0.5 mg/mL collagenase IV and 0.25 mg/mL DNaseI) at 37 ℃ for 45 minutes. The supernatant of the digestion solution was collected and processed through density gradient centrifugation in 80% and 40% isotonic Percoll solutions. After centrifugation, the cells at the interface between the two layers were collected, washed, and resuspended in PBS containing 2% FBS as the isolated lamina propria cells for further studies.

### Flow cytometry analysis

Flow cytometry analysis was performed to assess the immune cell populations in the colonic lamina propria, including macrophages (F4/80^+^CD11b^+^), monocytes (Ly6C^+^CD11b^+^), neutrophils (Ly6G^+^CD11b^+^), dendritic cells (CD11c^+^CD11b^+^), M1-type macrophages (CD80^+^F4/80^+^CD11b^+^), M2-type macrophages (CD206^+^F4/80^+^CD11b^+^), circulating inflammatory monocytes (Ly6C^hi^CX3CR1^low^CD11b^+^), and resident monocytes (Ly6C^low^CX3CR1^hi^CD11b^+^). The isolated cell suspensions were incubated with CD16/CD32 antibody for 10 min to block Fc receptors to reduce nonspecific binding of antibodies. They were then stained with a viability dye for 30 min, followed by incubating with a cocktail containing various fluorescent labeled antibodies for 30 min prior to fixation by 0.4% paraformaldehyde overnight at 4 ℃. The cells were washed, resuspended in PBS containing 2% FBS, and analyzed on a flow cytometer (LSR Fortessa, BD, San Jose, CA, USA). The FlowJo v10.8.1 software (TreeStar, Ashland, OR, USA) was applied for the data analysis. The antibody information for flow cytometry analysis was provided in **Table S4.**

### Construction of lentiviral expression plasmids

The lentiviral vector plasmid Plenti-C-mGFP was obtained from ORIGEN (PS100071, Rockville, MD, USA) with SgfI and MluI as the restriction sites. The genes of interest, N-HOIL-1, C-HOIL-1, WT-HOIL-1 and R/K-HOIL-1, were cloned based on the original plasmids expressing WT and R/K mutant HOIL-1 as the templates into the Plenti-C-mGFP plasmid, with a GFP tag linked at the C-terminal. This was done following the standard molecular cloning procedure of PCR reaction, DNA product purification, enzyme digestion, ligation and transformation. Similarly, the lentiviral vector plasmid pLVX-IRES-Hyg was purchased from Clontech (632185, Mountain View, CA, USA) with Xho1 and SpeI as restriction sites to construct the pLVX-IRES-Flag-C-HOIL-1-Hyg plasmid following the same procedure. The pLVX-IRES-Flag-C-HOIL-1-Mut-Hyg (C295A) plasmid was constructed using the site-directed mutagenesis strategy (NEB, M0491), with the wild-type Flag-C-HOIL-1-Hyg plasmid serving as the template. All target gene sequences of the constructed plasmids were confirmed by Sanger sequencing with primers listed in **Table S1**.

### Preparation of lentiviral particles

HEK-293T cells were cultured in DMEM medium supplemented with 10% FBS at 37 °C with 5% CO_2_. Cells were seeded into 10-cm culture dishes (6×10^6^ cells/dish) 1 day before transfection. The package plasmids (PAX8 and pVSVG) and expression plasmids of WT-HOIL-1, R/K-HOIL-1, C-HOIL-1 and C-HOIL-1-C295A were mixed with the PEI transfection reagent thoroughly. Cells were incubated with these mixtures at 37 °C and 5% CO_2_ for 6 h, and the culture medium was replaced with a fresh one. The medium was collected at 24 h and 48 h after transfection, followed by filtration (0.45 µm) and ultrahigh-speed centrifugation (27,000 rpm, Optimal-100XP, Beckman, Indianapolis, IN, USA) to harvest viral particles. These concentrated particles were re-suspended in ice cold PBS and stored at -80 ℃ for future use. The viral titer was quantified by measuring the P24 proteins using an enzyme-linked immunosorbent assay (ELISA) kit (Biodragon, BF06203, Suzhou, China) prior to the *in vivo* experiments.

### Gene editing in THP-1 cells by CRISPR-Cas9 approach

Human THP-1 monocytes were maintained in RPMI-1640 medium containing 10% FBS, 2 mM glutamine, and 1 mM sodium pyruvate in an incubator (Thermo-Fisher, Waltham, MA, USA) with 5% CO_2_ at 37 ℃.

The CRISPR-Cas9 gene editing technique was applied to construct HOIL-1 knockout (HOIL-1^-/-^) THP-1 cells. The sgRNAs (**Table S5**) targeting exon 3 and 6 of human *RBCK1* (encoding HOIL-1) were designed using the website (http://crispr.mit.edu), and the sequences were cloned into lentiCRISPRv2 vector (Addgene, #52961, Watertown, MA, USA). After sequence verification, the lentiCRISPRv2 recombinant plasmid and packaging plasmids were co-transfected into HEK-293T cells to obtain viral particles for infecting THP-1 cells.

THP-1 monocytes were treated with lentiviral particles containing different sgRNA sequences, and the infected cells were screened with puromycin. Single clones were selected, amplified, and verified by sequencing. Only clones with frameshift mutations, where the indels of the nucleotide base numbers were not a multiple of three, would they be chosen as established cell lines for further functional studies.

### Activation of different cellular signaling pathways in THP-1 cells

THP-1 cells and their genetically modified cells were seeded (1×10^6^ cells/well) in a 24-well plate and rested for 2 h before stimulation with human TNF-α (20 ng/mL) for different time periods. The activation of NF-κB was examined within a shorter period of 2 h, while the cytokine production was assessed at 24 h after stimulation. The R/K cells expressing either C-HOIL-1 or C-HOIL-1-C295A were stimulated with human TNF-α (50 ng/mL) for 30 min to evaluate the M1- and K48-linked ubiquitination; these cells were stimulated with human IFN-β (10 ng/mL) or human IFN-γ (100 ng/mL) for 0-2 h or for 24 h to examine STAT1 activation by immunoblotting or STAT1-mediated gene expression by RT-qPCR, respectively.

Similarly, R/K cells expressing either C-HOIL-1 or C-HOIL-1-C295A (1×10^6^ cells/well) were seeded into a 12-well plate and treated with PMA (50 ng/mL) for 24 h for cell differentiation into macrophages. After rinsing with PBS, they were rested for two days in a fresh medium. The differentiated cells were stimulated with human IFN-γ (20 ng/mL) and LPS (100 ng/mL) for 12 h or with human IL-4 (20 ng/mL) (MCE, HY-P70445, Monmouth Junction, NJ, USA) for 24 h to induce M1- or M2-type polarization, respectively. For NLRP3 inflammasome activation, the differentiated cells were stimulated with LPS (100 ng/mL) for 4 h, followed by ATP (5 mM) (Sigma-Aldrich, Louis, Missouri, USA) treatment for 30 min.

### Immunoblotting

The colon tissue lysates and cell lysates were prepared in a modified RIPA lysis buffer (50 mM Tris-HCl, 150 mM NaCl, pH 7.5) consisting of EGTA (2 mM), EDTA (2 mM), Triton X-100 (1%), and a Halt protease and phosphatase inhibitor cocktail. For colon tissues, a piece of tissue was first grounded with an electric tip grinder in the lysis buffer on ice for 20 min. After centrifugation at 13,000 rpm for 10 min at 4 ℃, the total protein concentration in the supernatant was quantified using the Bradford assay and adjusted to the same level. The proteins were boiled in a loading buffer for 5 min, separated by 10% SDS-PAGE, and transferred onto a PVDF membrane (Millipore, IPVH00010, Co. Cork, Ireland). The membrane was blocked with 2% BSA for 1 h prior to the incubation of primary antibodies overnight at 4 ℃; it was then blotted with fluorophore- or HRP-labeled secondary antibodies at room temperature for about 1 h. The fluorescence or chemiluminescence signals of the protein bands were detected on a ChemDoc^TM^ MP imaging system (Bio-Rad, Hercules, CA, USA). Similarly, cell lysates were prepared following the same procedure described above. The antibody information for immunoblotting was listed in **Table S4**.

### Immunoprecipitation and mass spectrometry analysis

The commercial FLAG magnetic beads or Protein A/G magnetic beads were mixed with cell lysates on a rotator overnight at 4 °C. The magnetic beads were then washed three times with washing buffer, and the bound proteins were extracted by elution buffer. The extracted proteins were separated by SDS-PAGE and probed by silver staining (Thermo-Fisher, #24612, Rockford, IL, USA). The protein bands of interest were cut and processed for identification by mass spectrometry. The Gene Ontology (GO) and KEGG enrichment analyses were performed using clusterProfiler (v4.18.4) in R (v4.5.2) with org.Hs.eg.db (v3.22.0) for gene ID conversion; visualization was generated by enrichplot (v1.30.4) and ggplot2 (v4.0.1) with additional data processing using dplyr, stringr, tidyr, patchwork, and scales. The targeted proteins of interest were validated by co-immunoprecipitation and immunoblotting.

TUBE2 magnetic beads were utilized to assess ubiquitination levels in the cells. To prevent the degradation of the polyubiquitin chains on the proteins, PR-619 and 1,10 phenanthroline were added to the cell lysates to inhibit deubiquitinases and ubiquitin-like isopeptidases, respectively. The lysates were mixed with the magnetic beads and processed with the same immunoblotting procedure as described above.

### Immunofluorescence staining

To examine the immune cell infiltration in the colon by immunofluorescence imaging, the dewaxed and re-hydrated colon tissue sections (4 µm thick) were first processed for antigen retrieval, and then blocked and incubated with specific primary antibodies (1:800 dilution) for 12 h in a humidified dish at 4 ℃. Different immune cells were identified by specific markers: CD11b^+^ for myeloid cells, F4/80^+^ for macrophages, MPO^+^ for neutrophils, CD4^+^ for T helper cells, F4/80^+^iNOS^+^ for M1-type macrophages, and F4/80^+^CD163^+^ for M2-type macrophages. They were then incubated with fluorophore-labeled secondary antibodies (1:1800 dilution) for 7 h at 4 ℃, followed by 10-min DAPI staining at room temperature; the fluorescence images of these sections were captured on an Olympus fluorescence microscope (model BX51, Tokyo, Japan).

For immunofluorescence analysis on THP-1 cells (R/K-HOIL-1 cells expressing FLAG-C-HOIL-1), these cells were fixed with 4% paraformaldehyde and spun onto a glass slide using a Cytospin (Cytospin4, Thermo Fisher, Runcorn, UK) at 800×g for 5 min. Subsequently, they were permeabilized with 0.2% Triton X-100 for 10 min, blocked with 5% BSA for 1 h, and incubated with primary antibodies against FLAG (for C-HOIL-1) and STAT1 overnight at 4 ℃. Cells were then incubated with fluorophore-labelled secondary antibodies (Alexa Fluor 568 for FLAG and Alexa Fluor 647 for STAT1) for 1.5 h, and stained with Hoechst 33258 (for the nucleus) for 3 min at the room temperature. The fluorescence images were acquired on a confocal microscope (LSM-800, Carl Zeiss AG, Oberkochen, Germany). The information of all primary and secondary antibodies used for immunofluorescence was provided in **Table S4**.

### Real-time quantitative PCR

A piece of the colon tissue (50 to 100 mg) was mixed with 1 mL TRIZOL and homogenized using a motorized grinder. These homogenates were centrifuged at 12,000 rpm for 10 min at 4 ℃, and the supernatants were collected for RNA extraction. Total RNA was isolated by a serial procedure of chloroform separation, isopropanol precipitation, and ethanol washing. The extracted RNA was converted to cDNA using a reverse transcription kit (Accurate Biology, AG11706, Changsha, China). The genes of interest were amplified with specific primers and SYBR Green Master Mix (GenStar, A314, Suzhou, China) on a real-time PCR instrument (QuantStudio 3, Applied Biosystems, Singapore). The 2^-ΔΔCT^ approach was used to estimate the relative mRNA expression. All primer sequences were listed in **Table S1**.

### Luminex and enzyme-linked immunosorbent assay

Cytokines in the colon tissue lysates were quantified using multiplex protein quantitative analysis (Luminex, Universal Biotechnology, Shanghai, China). Twenty three cytokines were analyzed, including IL-1α, IL-1β, IL-2, IL-3, IL-4, IL-5, IL-6, IL-9, IL-10, IL-12p40, IL-12p70, IL-13, IL-17A, Eotaxin, G-CSF, GM-CSF, IFN-γ, CXCL1/KC, CCL2/MCP-1, CCL3/MIP-1α, CCL4/MIP-1β, RANTES/CCL5, and TNF-α. These cytokine levels were processed using Heml 1.0 software to generate a heat map for comparison among different groups. Selected cytokines of IL-1β, IL-6, CXCL1/KC, TNF-α, CCL2/MCP-1, CCL4/MIP-1β, IL-10, IL-5, and IL-17A were validated by ELISA. Cytokine levels of IL-8 and CCL4/MIP-1β in THP-1 cell culture medium were examined using ELISA kits according to the manufacturer's instruction.

### Statistical analysis

The data was analyzed and graphed using GraphPad Prism 9 software. All results were displayed as mean ± standard error of the mean (SEM). The student t-test was used for comparing two groups, whereas one- or two-way ANOVA with Bonferroni post-hoc tests were applied for multiple comparisons whenever applicable. The differences were considered significant when p < 0.05 with a p value specified on the plot.

## Results

### Inability to cleave HOIL-1 exacerbated DSS-induced colitis in mice

We previously discovered that HOIL-1 cleavage by MALT1 down-regulates the transcription factor NF-κB signaling 13. Such a negative regulatory role of HOIL-1 cleavage was further verified in HOIL-1 deficient human fibroblasts that express uncleavable HOIL-1 (i.e., the cleavage site of arginine 165 was replaced with lysine as HOIL-1-R165K, or HOIL-1-R/K) 16. In these modified fibroblasts, the defect in HOIL-1 cleavage results in the u p-regulation of inflammatory genes and signaling pathways, including Toll-like receptor (TLR), IFN, IL-1, and chemokine signaling pathways. These findings suggest that HOIL-1 cleavage is essential for regulating NF-κB signaling and inflammatory responses *in vitro*.

**Figure 1:**
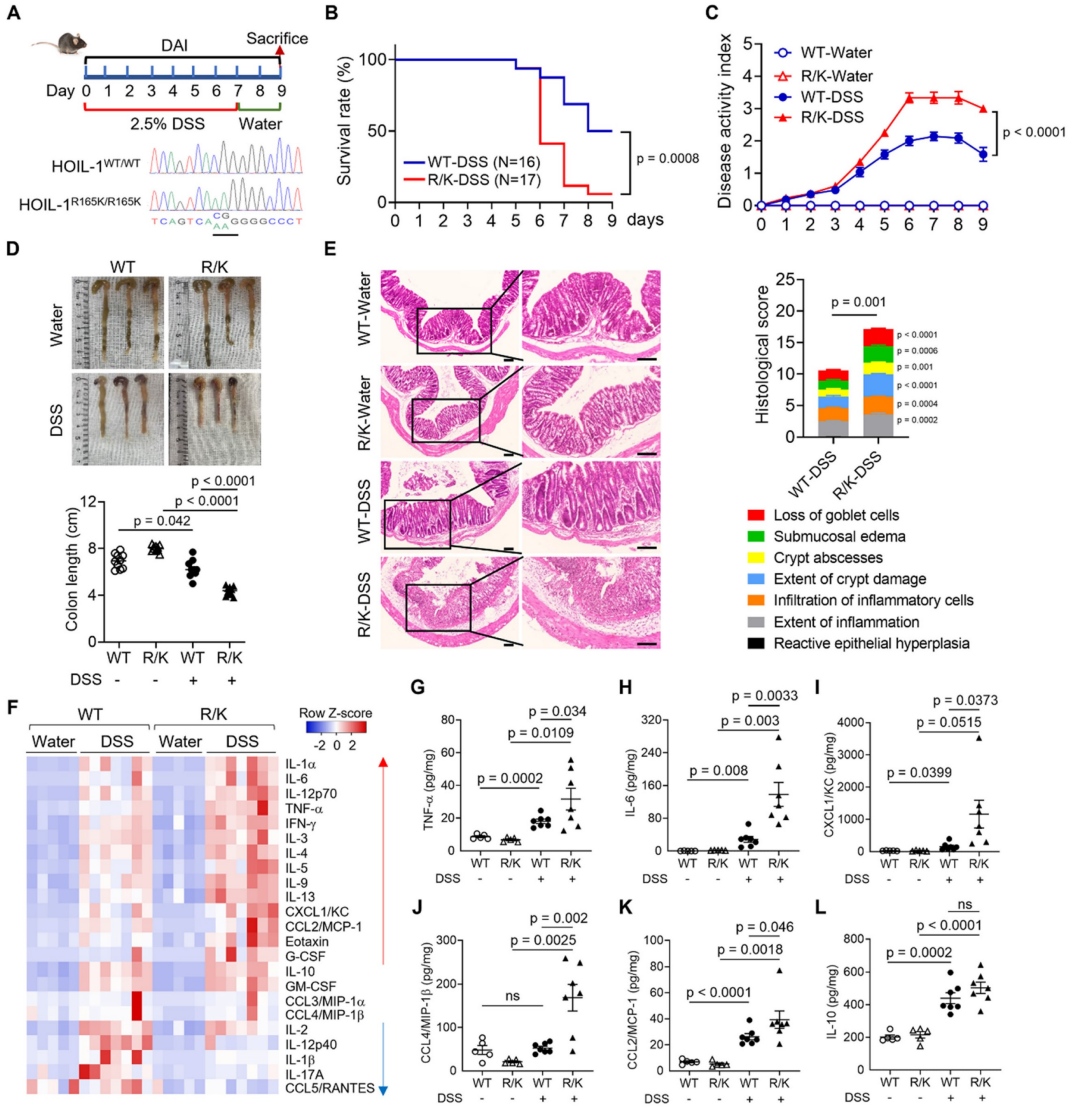
** Defect in HOIL-1 cleavage aggravates DSS-induced colitis in mice.** (**A**) A schematic diagram showing the DSS-induced colitis model in HOIL-1-R/K mice vs. WT mice with four experimental groups: WT-water, WT-DSS, R/K-water, R/K-DSS; histograms of the DNA sequences of HOIL-1 confirming the homozygous genotypes of WT HOIL-1 (HOIL-1^WT/WT^; R165: CGG) and HOIL-1-R165K (HOIL-1^RK/RK^; K165: AAG) in mice by Sanger sequencing (bottom). (**B-E**) The survival analysis (**B**), the disease activity index (DAI) (**C**), the colon length (**D**), and the H&E staining (left) with the histological scores (right) (**E**) of WT and R/K mice with/without DSS-induced colitis; DSS: 2.5%; scale bar = 100 µm; N = 5 for water groups, N = 16 for WT-DSS group, N = 17 for R/K-DSS group in (B, C); N = 10 in (D); N = 11 for WT-DSS group, N = 7 for R/K-DSS group in (E). (**F**) The heatmap of the normalized levels of 23 cytokines by Luminex in the colon tissues of WT and R/K mice with/without DSS-induced colitis; N = 5 for water groups, N = 7 for DSS groups. (**G-L**) The levels of selected cytokines TNF-α (**G**), IL-6 (**H**), CXCL1/KC (**I**), CCL4/MIP-1β (**J**), CCL2/MCP-1 (**K**) and IL-10 (**L**) in the colon tissues measured by ELISA; N = 5 for water groups, N = 7 for DSS groups. ns: not significant.

To further define the role of HOIL-1 cleavage *in vivo*, we established an uncleavable HOIL-1 knock-in (HOIL-1^R165K/R165K^) mouse model. The homologous HOIL-1^R165K/R165K^ mice were designated as R/K mice and were compared with the wild-type (WT) littermates. The genotypes of the in-house bred WT and R/K mice were validated by Sanger sequencing (**Figure 1A**) prior to all experiments. HOIL-1 cleavage in the splenic mononuclear cells of these mice was examined by immunoblotting; the C-terminal fragment of HOIL-1 cleavage (C-HOIL-1) was clearly observed under PMA/ionomycin stimulation over time in these cells with WT-HOIL-1, while it was absent in those with R/K-HOIL-1 (**Figure S1A**). In addition, the PMA/ionomycin-induced C-HOIL-1 production was diminished under the treatment of MALT1 protease inhibitor MLT-748 (**Figure S1B**). Such a defect also promoted NF-κB activation (**Figure S1A**), increased the total linear ubiquitination (**Figure S1C**), and up-regulated inflammatory gene expression, including *Tnfa, Il6, Il1b, Il12b, Ccl2, Ccl4* and *Cxcl1* under PMA/ionomycin stimulation when compared with WT-HOIL-1 (**Figure S1D-J**). These results recapitulated our previous findings in human fibroblasts that uncleavable HOIL-1 exacerbates inflammatory responses.

However, the physiological role of HOIL-1 cleavage in regulating intestinal inflammation remained elusive. To unveil this puzzle, a classical (2.5%) DSS-induced colitis mouse model was employed on WT and R/K mice to mimic ulcerative colitis of IBD (**Figure 1A**). It was found that the R/K mice exhibited a lower survival rate (**Figure 1B**), a higher disease activity index (DAI) (**Figure 1C**), and a shorter colon length (**Figure 1D**) when compared with the WT mice. The histological analysis on the colon tissues revealed a more inflamed and damaged phenotype with features of loss of goblet cells, submucosal edema, crypt abscesses, extent of crypt damage, and infiltration of inflammatory cells in the R/K mice than in the WT mice (**Figure 1E**). Using the 23-plex Luminex assay for measuring the cytokine levels in the mouse colon tissues, we found a clear pattern of many up-regulated pro-inflammatory cytokines and chemokines in the R/K group when compared with the WT group upon DSS induction (**Figure 1F**); several up-regulated cytokines/chemokines were validated by ELISA, including TNF-α, IL-6, CXCL1/KC, CCL4/MIP-1β, and CCL2/MCP-1 (**Figure 1G-K**). Notably, there was no difference in the levels of the anti-inflammatory cytokine IL-10 (**Figure 1L**), IL-17A for Th17 response (**Figure S2A**), or IL-5 for Th2 response (**Figure S2B**) between the R/K and WT mice. These results demonstrated that the defect in HOIL-1 cleavage exacerbates DSS-induced colitis in mice.

### The defect in HOIL-1 cleavage promoted inflammatory myeloid cell infiltration to the colon

**Figure 2:**
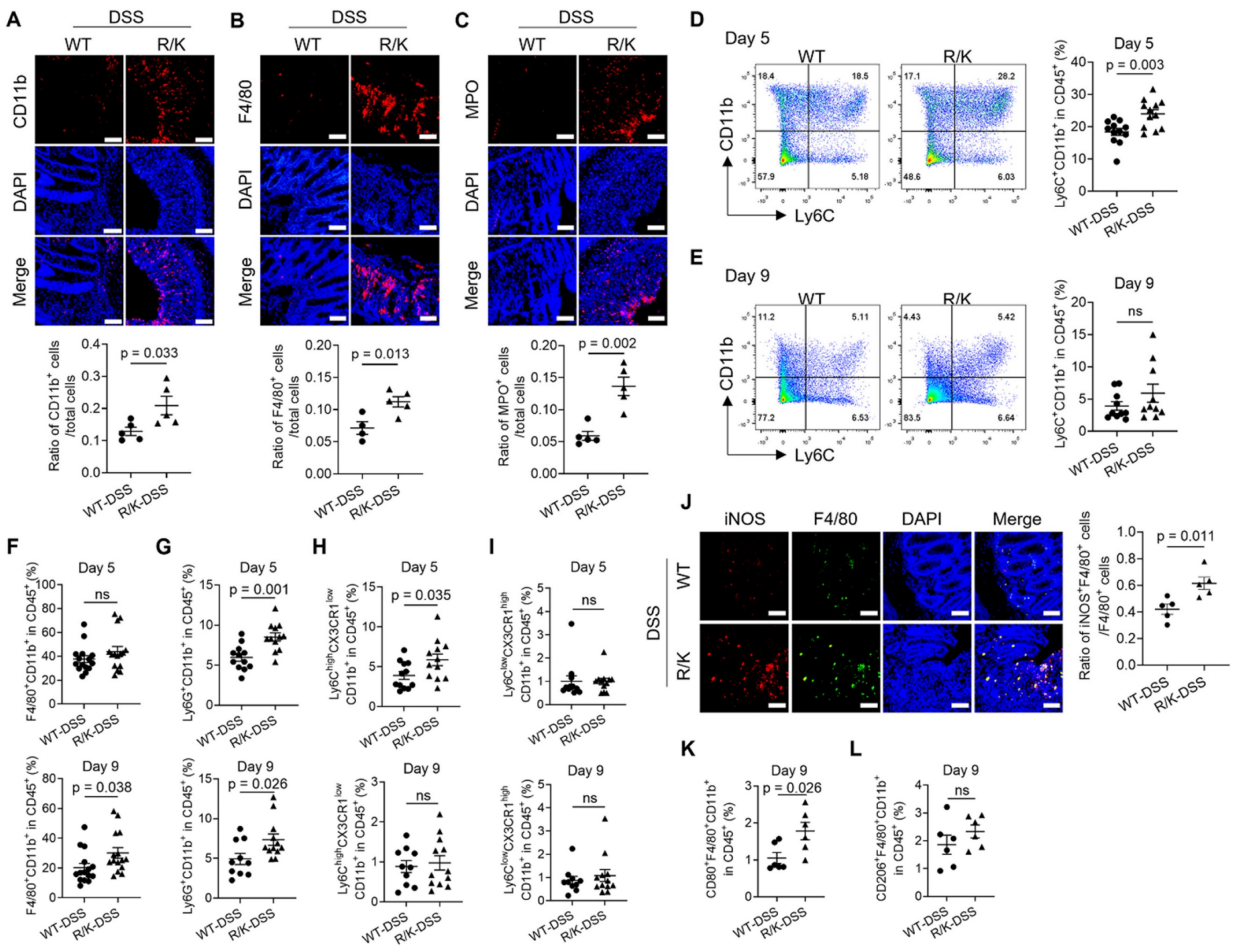
** Uncleavable HOIL-1 increases the infiltration of inflammatory myeloid cells in the colon of R/K mice with DSS-induced colitis.** (**A-C**) Immunofluorescence images showing the increased percentage of CD11b^+^ myeloid cells (**A**), F4/80^+^ macrophages (**B**) and MPO^+^ neutrophils (**C**) in the colon tissue of R/K mice with DSS-induced colitis; the immune cells were indicated by red color while the cell nucleus was stained in blue (by DAPI); the quantitative analysis was shown at the bottom; scale bar = 100 µm; N = 5. (**D, E**) The percentage of Ly6C^+^CD11b^+^ monocytes in the colonic lamina propria of WT and R/K mice upon DSS induction at Day 5 (**D**) and Day 9 (**E**) by flow cytometry analysis; N = 12 for Day 5, N = 10 for Day 9. (**F-I**) Multi-color flow cytometry analysis on the percentage of F4/80^+^CD11b^+^ macrophages (**F**), Ly6G^+^CD11b^+^ neutrophils (**G**), Ly6C^hi^CX3CR1^low^CD11b^+^ circulating monocytes (**H**), and Ly6C^low^CX3CR1^hi^CD11b^+^ tissue resident monocytes (**I**) in the colonic lamina propria of WT and R/K mice with DSS-induced colitis at Day 5 (top) and Day 9 (bottom); N = 15 in (F); N = 12 for Day 5, N = 10 for WT-DSS group and N = 12 for R/K-DSS group for Day 9 in (G-I). (**J**) Immunofluorescence images showing the percentage of F4/80^+^iNOS^+^ M1-type macrophages in the colon tissues of WT and R/K mice upon DSS induction (Day 9) with quantitative analysis on the right; the iNOS and F4/80 were stained in red and green, respectively, while the cell nucleus was stained in blue (by DAPI); scale bar = 100 µm; N = 5. (**K, L**) Flow cytometry analysis on the percentage of CD80^+^F4/80^+^CD11b^+^ M1-type macrophages (**K**) and CD206^+^F4/80^+^CD11b^+^ M2-type macrophages (**L**) in the colonic lamina propria of WT and R/K mice upon DSS induction on Day 9; N = 6. ns: not significant.

It is known that the recruitment of myeloid cells, such monocytes, neutrophiles, and macrophages, to the colon contributes to the pathogenesis of gut inflammation in IBD 23-25. HOIL-1 deficient patients also present episodes of gastrointestinal inflammation with hyper-activated peripheral mononuclear cells 9, 10. Based on these facts, we next investigated the impact of uncleavable HOIL-1 on the immune cell infiltration in the colon during colitis. The immune cells of interest in the colon were identified by their typical markers: CD11b^+^ for myeloid cells, F4/80^+^ for macrophages, MPO^+^ for neutrophils, and CD4^+^ for T helper lymphocytes. By immunofluorescence imaging on the colon tissues, we found that the percentages of CD11b^+^ (**Figure 2A**), F4/80^+^ (**Figure 2B**), and MPO^+^ (**Figure 2C**) cells were significantly higher in the R/K mice than in the WT mice. However, no difference was observed in the percentage of CD4^+^ T cells between R/K and WT groups (**Figure S3**). These results suggested that the increased myeloid cell infiltration to the colon may significantly contribute to the observed hyper severity of DSS-induced colitis in the R/K mice.

Next, we utilized flow cytometry analysis to investigate the dynamic changes of infiltrated myeloid cells in the colonic lamina propria on Day 5 (onset of colitis symptoms) and Day 9 (end of the colitis model) of the colitis model. Different immune cell types were defined using the gating strategy shown in **Figure S4A and B**. The analysis revealed distinct dynamic profiles of the infiltrated myeloid cells. First, the percentage of Ly6C^+^CD11b^+^ monocytes in total leukocytes (CD45^+^ cells) was significantly increased in the R/K mice on Day 5, but not on Day 9, when compared with the WT ones (**Figure 2D and E**). Conversely, the percentage of F4/80^+^CD11b^+^ macrophages was significantly higher in the R/K mice than in the WT mice on Day 9, but not on Day 5 (**Figure 2F**). Different from the above two profiles, the percentage of Ly6G^+^CD11b^+^ neutrophils was elevated in the R/K mice on both Day 5 and Day 9 when compared with the WT mice (**Figure 2G**). Note that the percentage of classical dendritic cells (cDC, CD11c^+^CD11b^+^) remained unchanged in the colonic lamina propria of WT and R/K mice during colitis (**Figure S4C**). These results suggest that during the development of colitis, the defect in HOIL-1 cleavage promotes the monocyte infiltration earlier, and affects the macrophage population later in the colonic lamina propria, while the neutrophil infiltration is continuously elevated in R/K mice throughout the model period as indicator of intestinal inflammation. This also indicated an essential role of monocytes and macrophages in the increased intestinal inflammatory responses of the R/K mice.

Inspired by these findings, we speculated that the recruitment and differentiation of inflammatory monocytes into macrophages in the colon may drive the aggravation of colitis in the R/K mice. To test this hypothesis, we examined the dynamic profiles of the circulating monocytes (Ly6C^hi^CX3CR1^low^) known for participating in the inflammatory responses and the tissue-resident monocytes (Ly6C^low^CX3CR1^hi^) responsible for maintaining intestinal immune homeostasis 26 during colitis. Intriguingly, the circulating monocytes in the lamina propria of the R/K mice were significantly increased on Day 5, but not on Day 9 when compared with the WT mice (**Figure 2H**); however, no difference was observed in the percentage of the tissue-resident monocytes between the R/K and WT mice (**Figure 2I**). The increase in the circulating inflammatory monocytes in the colon at the early phase of colitis may be the key driver for the excessive intestinal inflammation in the R/K mice.

In addition to inflammatory monocyte infiltration, we also wondered if the defect in HOIL-1 cleavage imbalanced the phenotypes of macrophages in the colon, contributing to the exacerbation of colitis. The immunofluorescence images revealed that the percentage of iNOS^+^ inflammatory M1-type macrophages in the colon was significantly higher in the R/K mice than in the WT mice on Day 9 after DSS induction (**Figure 2J**), but the percentage of CD163^+^ anti-inflammatory M2-type macrophages remained similar between the WT-DSS and R/K-DSS groups (**Figure S5A**). This finding was recapitulated in the flow cytometry analysis with a significant difference found in the percentage of CD80^+^ M1 macrophages (**Figure 2K**) but not in that of CD206^+^ M2 macrophages (**Figure 2L**) in the colonic lamina propria on Day 9 between the WT and R/K groups. Such a difference was not seen at the early phase of colitis (Day 5) (**Figure S5B**). Collectively, these results demonstrated that the defect in HOIL-1 cleavage exacerbates intestinal inflammation by dynamically recruiting more inflammatory monocytes, neutrophils, and M1-type macrophages to the colon, leading to increased severity of DSS-induced colitis.

### Uncleavable HOIL-1 enhanced NF-κB activation and inflammatory responses in THP-1 monocytes

**Figure 3:**
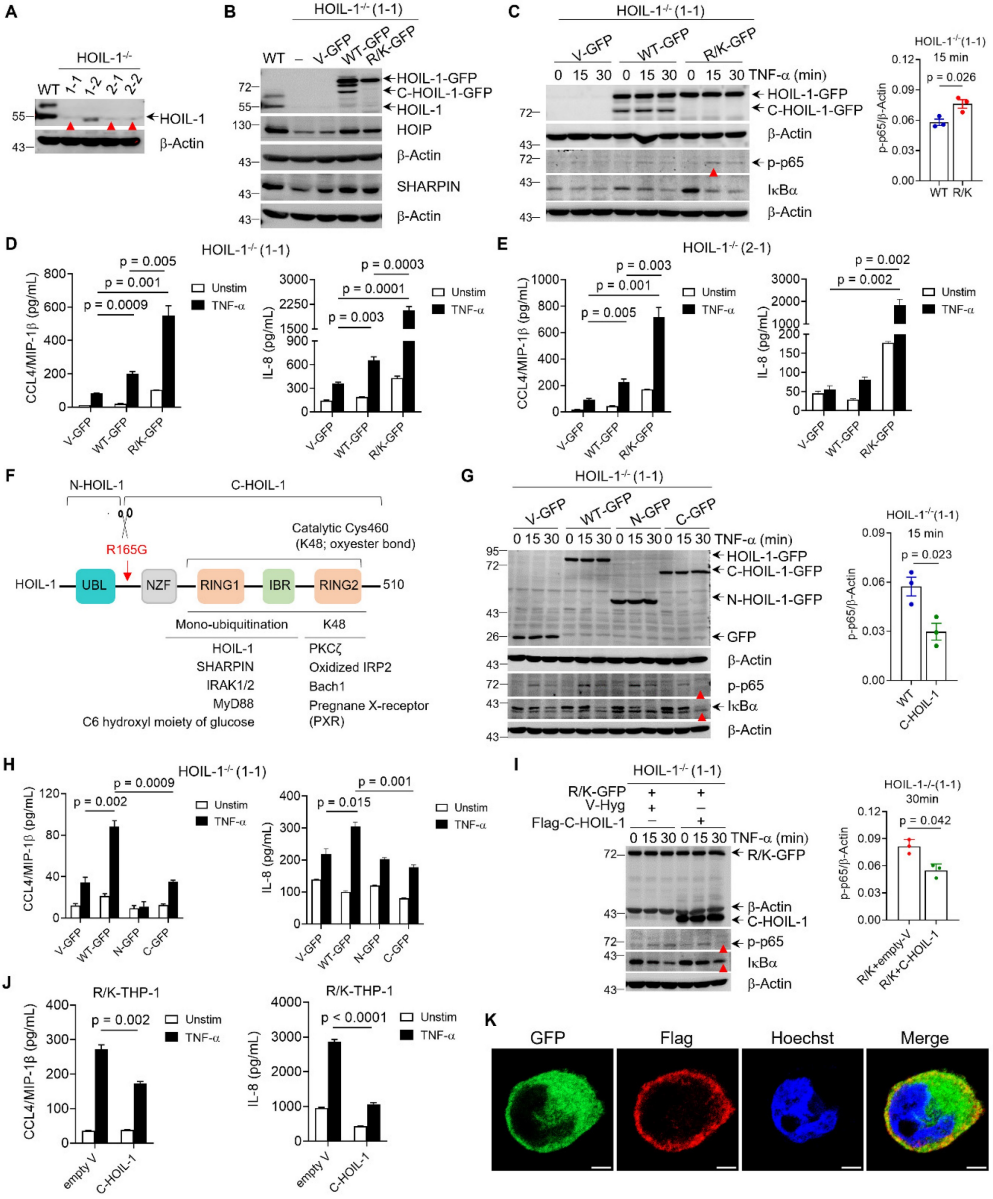
** The C-HOIL-1 fragment of HOIL-1 cleavage is biologically functional in regulating NF-κB activation and inflammatory responses in THP-1 cells.** (**A**) Immunoblots showing the expression of HOIL-1 protein in selected clones (1-1, 1-2, 2-1 and 2-2) of constructed HOIL-1^-/-^ THP-1 cells by CRISPR/Cas9 gene editing technique; red arrows indicated the absence of HOIL-1 protein band; β-Actin as the internal control. (**B**) Immunoblots showing HOIL-1 cleavage producing the fragment C-HOIL-1 in the HOIL-1^-/-^(1-1) cells expressing WT HOIL-1 (WT-GFP), but not in cells expressing uncleavable HOIL-1-R/K (R/K-GFP); expression of WT or R/K HOIL-1 restored the levels of HOIP and SHARPIN in HOIL-1^-/-^(1-1) cells; the WT THP-1 cells and HOIL-1^-/-^(1-1) cells transduced with empty vectors (V-GFP) as the controls; β-Actin as the internal control. (**C**) Immunoblots showing the phosphorylation of p65 and degradation of IκBα for NF-κB activation upon TNF-α (20 ng/mL) stimulation in R/K-GFP cells vs. WT-GFP cells with densitometry analysis on p-p65 on the right; β-Actin as the internal control. (**D, E**) The production of CCL4/MIP-1β (left) and IL-8 (right) in HOIL-1^-/-^(1-1) (**D**) and HOIL-1^-/-^(2-1) (**E**) cells expressing V-GFP, WT-GFP and R/K-GFP with or without TNF-α (20 ng/mL) stimulation for 24 h by ELISA; N = 3. (**F**) A schematic illustration of HOIL-1 structural domains and the known substrates of its E3 ligase domain (RING1-IBR-RING2, RBR); HOIL-1 cleavage by MALT1 at R165G to produce N- and C-terminal fragments (N-HOIL-1 and C-HOIL-1, respectively). (**G**) Immunoblots showing the phosphorylation of p65 and IκBα degradation in HOIL-1^-/-^(1-1) cells expressing WT-, N-, and C-HOIL-1 upon TNF-α (20 ng/mL) stimulation over time with quantitative analysis on p-p65 for C-HOIL-1 vs. WT-HOIL-1 at 15 min on the right; V-GFP as a control; immunoblotting GFP for HOIL-1 expression; β-Actin as the internal control. (**H**) The production of CCL4/MIP-1β (left) and IL-8 (right) in HOIL-1^-/-^(1-1) expressing none (V-GFP), WT-HOIL-1 (WT-GFP), N-HOIL-1 (N-GFP), and C-HOIL-1 (C-GFP) with or without TNF-α (20 ng/mL) stimulation for 24 h by ELISA; N = 3. (**I**) Immunoblots showing NF-κB activation (p-p65 and IκBα) upon TNF-α (20 ng/mL) stimulation over time in R/K-GFP cells expressing V-Hyg (empty) or Flag-C-HOIL-1 with quantitative analysis on p-p65 levels at 30 min on the right; β-Actin as the internal control. (**J**) The production of CCL4/MIP-1β (left) and IL-8 (right) in R/K-GFP cells expressing V-Hyg or Flag-C-HOIL-1 with or without TNF-α (20 ng/mL) stimulation for 24 h by ELISA; N = 3. (**K**) The confocal images demonstrating the intracellular localization of C-HOIL-1 in R/K-GFP cells expressing Flag-tagged C-HOIL-1; the GFP and Flag were stained in green and red, respectively; the nucleus was stained with Hoechst (in blue); scale bar = 5 µm; N = 3.

Based on our *in vivo* results, we hypothesize that the defect in HOIL-1 cleavage drives the myeloid inflammatory responses, particularly in monocytes and macrophages. To test this hypothesis, we genetically engineered the human THP-1 monocytic cells that expressed HOIL-1-R/K for *in vitro* studies. The CRISPR-Cas9 approach was employed to first establish HOIL-1^-/-^ THP-1 cells by targeting the exon 3 and exon 6 of *RBCK1* (encoding HOIL-1). Two sgRNAs targeting exon 3 effectively reduced the HOIL-1 protein level (**Figure S6A**), and the knockout efficiency was further improved by selecting two single clones (1-1 and 2-1) from each (**Figure 3A**) for subsequent *in vitro* experiments. Compared with the WT cells, the absence of HOIL-1 resulted in impaired NF-κB activation upon TNF-α stimulation (**Figure S6B**), which was consistent with the findings in HOIL-1 deficient human peripheral mononuclear cells and fibroblasts 9.

Next, the lentiviral transduction approach was used to establish the stable expression of green fluorescence protein (GFP)-tagged WT-HOIL-1 (WT-GFP) or HOIL-1-R/K-GFP (R/K-GFP) in these two HOIL-1^-/-^ clones to assess the effect of HOIL-1 cleavage on NF-κB activation. The GFP^+^ cells were sorted out to ensure the equal level of WT-HOIL-1 and R/K-HOIL-1 for comparison. The presence and absence of C-HOIL-1 bands in the immunoblotting results confirmed the successful establishment of WT-GFP and R/K-GFP expression in HOIL-1^-/-^ THP-1 monocytes, respectively (**Figure 3B and C**). By treating these cells with the MALT1 inhibitors MI-2 and MLT-748, the C-HOIL-1 bands were reduced (**Figure S7**), confirming that HOIL-1 cleavage is dependent on the MALT1 protease activity. When compared with WT-GFP, the R/K-GFP exhibited stronger activation of NF-κB pathway (**Figure 3C, Figure S8**) and enhanced production of the pro-inflammatory chemokines IL-8 and CCL4/MIP-1β (**Figure 3D and E**) upon TNF-α stimulation. These results demonstrated that the defect in HOIL-1 cleavage up-regulates TNF-α-mediated NF-κB signaling and promotes the inflammatory responses in monocytes.

### The C-terminal fragment resulted from HOIL-1 cleavage down-regulated NF-κB signaling and pro-inflammatory cytokine production

We previously identified that HOIL-1 is cleaved at the residue of R165, resulting in the formation of a smaller N-terminal fragment (N-HOIL-1) and a larger C-terminal one (C-HOIL-1) (**Figure 3F**) 13. While N-HOIL-1 containing the ubiquitin-like (UBL) domain keeps the ability to interact with HOIP and SHARPIN, C-HOIL-1 consisting of a Npl4 zinc finger (NZF) domain and an E3 catalyzing RING1-IBR-RING2 (RBR) domain is released from LUBAC 13. The intracellular persistence of C-HOIL-1 and its RBR structure raised our strong interests in uncovering the unknown functions of C-HOIL-1 in regulating inflammatory signaling. To unveil this puzzle, we first established the stable expression of GFP-tagged N-HOIL-1 (N-GFP) and C-HOIL-1 (C-GFP) in HOIL-1^-/-^ THP-1 cells. It was found that the expression of N-HOIL-1 restored the protein levels of endogenous HOIP and SHARPIN in HOIL-1^-/-^ cells similar to WT-HOIL-1 (**Figure S9A**), indicating that N-HOIL-1 retained the ability to interact with HOIP and SHARPIN to stabilize the LUBAC structure 11. In contrast, the decreased HOIP and SHARPIN levels were not reversed by the expression of C-HOIL-1, simply because C-HOIL-1 did not bind to either HOIP or SHARPIN (**Figure S9B**) owing to the absence of UBL domain in C-HOIL-1. As a result, C-HOIL-1 is released from LUBAC.

To investigate whether the released C-HOIL-1 possesses distinct biological functions, we first characterized the effects of C-HOIL-1 on TNF-α-mediated signaling in HOIL-1^-/-^ THP-1 cells. The expression of C-HOIL-1 significantly inhibited NF-κB activation (**Figure 3G**) and reduced the production of IL-8 and CCL4 (**Figure 3H**) upon TNF-α stimulation when compared with WT-HOIL-1. We then further established stable expression of C-HOIL-1 (Flag-C-HOIL-1) in R/K-GFP cells, which lack C-HOIL-1. We found that the restoration of C-HOIL-1 was able to reverse the TNF-α-triggered hyper-activation of NF-κB (**Figure 3I**) and increased production of CCL4 and IL-8 (**Figure 3J**) observed in the R/K condition. These results, for the first time, demonstrated that the resulting C-HOIL-1 fragment of HOIL-1 cleavage is capable of regulating TNF-α-mediated inflammatory signaling in THP-1 monocytes. Finally, we employed the immunofluorescence imaging technique to examine the intracellular localization of C-HOIL-1. Notably, C-HOIL-1 (Flag labeled) predominantly accumulated at the cytoplasmic periphery near the cell membrane, very different from R/K-HOIL-1 (GFP labeled) that was dispersed in the cytoplasm (**Figure 3K**). Such a unique subcellular distribution pattern also strongly suggested that C-HOIL-1 may have distinct functions different from the full-length HOIL-1.

### C-HOIL-1 interacted with STAT1 and down-regulated interferon stimulated gene expression

**Figure 4:**
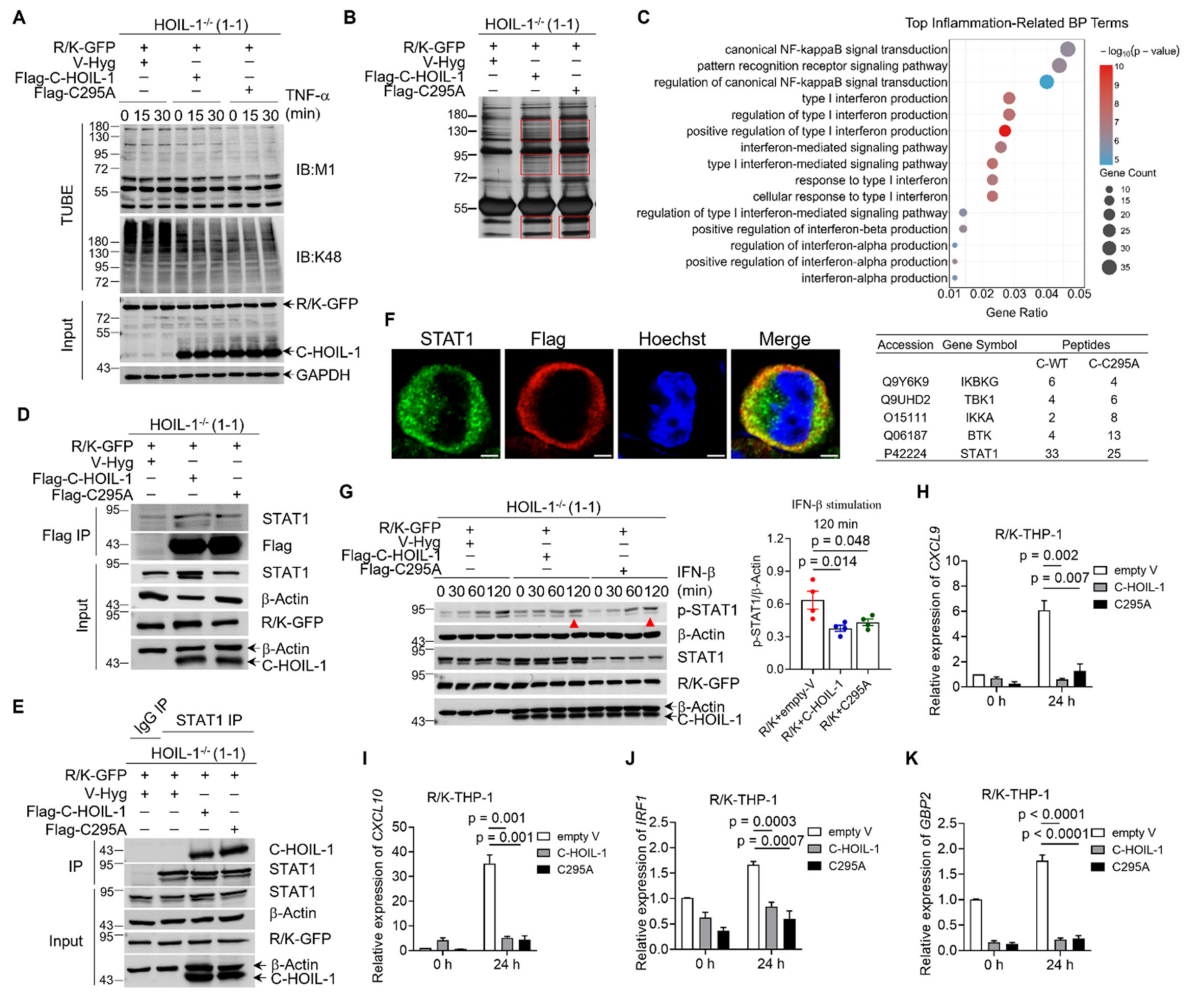
** C-HOIL-1 interacts with STAT1 and down-regulates IFN-β-STAT1 signaling.** (**A**) Immunoblots of linear (M1-linked) and K48-linked polyubiquitin chains in R/K-GFP cells expressing V-Hyg, Flag-C-HOIL-1 or Flag-C-HOIL-1-C295A (enzymatically inactive) upon TNF-α (20 ng/mL) stimulation over time; GAPDH as the internal control. (**B**) Silver staining on the Flag enriched total proteins of R/K-GFP cells expressing V-Hyg, Flag-C-HOIL-1, or Flag-C295A; the differentially enriched proteins bands (vs. V-Hyg group) were identified (red rectangular) and processed for mass spectrometry. (**C**) The bubble plot displaying the top 15 inflammation-related biological processes identified from Gene Ontology (GO) analysis of proteins enriched in the Flag-C-HOIL-1 and Flag-C295A groups; the data information of the proteins of interest was listed in a table at the bottom. (**D**) The co-immunoprecipitation assay using Flag magnetic beads to pull-down STAT1 in R/K-GFP cells expressing Flag-C-HOIL-1 or Flag-C295A; β-Actin as the internal control. (**E**) The co-immunoprecipitation assay using anti-STAT1 antibody-bound protein A/G magnetic beads to pull-down Flag-C-HOIL-1 or Flag-C295A in R/K-GFP cells; β-Actin as the internal control. (**F**) The confocal images showing the co-localization (yellow color in the merged images) of Flag-C-HOIL-1 (red) with endogenous STAT1 (green) in R/K-GFP cells; the nucleus was stained by Hoechst in blue; scale bar = 5 µm. (**G**) Immunoblots showing the changes in the phosphorylation of STAT1 (p-STAT1) upon IFN-β (10 ng/mL) stimulation over time in R/K-GFP cells expressing V-Hyg, Flag-C-HOIL-1, or Flag-C295A with quantitative analysis on p-STAT1 at 120 min right the right; β-Actin as the internal control. (**H-K**) The relative expressions of STAT1 controlled genes *CXCL9* (**H**), *CXCL10* (**I**), *IRF1* (**J**), and *GBP2* (**K**) in R/K-GFP cells expressing V-Hyg, Flag-C-HOIL-1, or Flag-C295A upon IFN-β (100 ng/mL) stimulation for 24 h by RT-qPCR; N = 3.

Beyond its scaffolding function in LUBAC, HOIL-1 is also an E3 ligase capable of catalyzing mono-ubiquitination or K48-linked polyubiquitination through the RBR domain (**Figure 3F**) 27-32. Recently, a catalytic site of HOIL-1 in the RBR domain was identified at cysteine 460 (Cys460) in the RING2 region, with the ability to add mono-ubiquitin on HOIL-1 to down-regulate LUBAC-mediated linear ubiquitination 28. Since C-HOIL-1 contains the RBR domain, we rationalized that this catalytic site (Cys460 of HOIL-1 or Cys295 of C-HOIL-1) may contribute to the distinct function of C-HOIL-1. Accordingly, we constructed an RBR inactive C-HOIL-1 (Flag-C295A) to investigate the impact of the E3 activity on regulating the NF-κB signaling pathway. Surprisingly, the C295A mutated C-HOIL-1 exhibited a similar inhibitory activity on NF-κB signaling and IL-8 production when compared with wild-type C-HOIL-1 (**Figure S10**). On the other hand, a difference in down-regulating the total M1- (linear) and K48-linked ubiquitination was observed between the mutated and wild-type C-HOIL-1 in R/K-GFP cells upon TNF-α stimulation (**Figure 4A**). This suggested that the RBR function of C-HOIL-1 may participate in novel signaling pathways other than LUBAC-mediated NF-κB activation.

In order to identify the novel pathways that are regulated by C-HOIL-1, magnetic beads were applied to enrich Flag-C-HOIL-1 and Flag-C295A as well as their interacting proteins in R/K-GFP cells. The differential protein bands in each group (framed by the red boxes) (**Figure 4B**) were harvested and processed for mass spectrometry analysis to identify potential interacting proteins with C-HOIL-1. Based on the mass spectrometry data, we conducted Gene Ontology (GO) and Kyoto Encyclopedia of Genes and Genomes (KEGG) analyses on the enriched proteins from both Flag-C-HOIL-1 and Flag-C295A groups. The top 15 enriched KEGG pathways were presented in a bar graph (**Figure S11A**), and the top 15 inflammation-associated biological processes were shown in an adjoining bubble plot (**Figure 4C**). These biological processes were designated as NF-κB, pattern recognition receptor, or type-I IFN signaling pathways, in which five identified proteins of interest were listed below the chart (**Figure 4C**). Among them, signal transducer and activator of transcription 1 (STAT1) was confirmed as a binding target of C-HOIL-1 by co-immunoprecipitation with Flag-C-HOIL-1 or Flag-C295A (**Figure 4D**) and with the endogenous STAT1 (**Figure 4E**). Other potential targets, including inhibitor of nuclear factor kappa-B kinase subunit alpha (IKKα), IKKγ (or NEMO), and Bruton's tyrosine kinase (BTK), were not enriched by either Flag-C-HOIL-1 or Flag-C295A (**Figure S11B-D**). A direct physical interaction between C-HOIL-1 and STAT1 was further validated by the pull-down experiments on recombinant GST-tagged STAT1 and His-tagged C-HOIL-1 proteins in a test tube (**Figure S12**). Furthermore, we observed the co-localization of Flag-C-HOIL-1 (red) with STAT1 (green) at the cytoplasmic periphery using the immunofluorescence imaging (**Figure 4F**). These results demonstrated that C-HOIL-1 directly binds to STAT1.

STAT1 is known as an essential transcription factor that can be activated by various cytokines and IFNs to drive expression of interferon-stimulated genes (ISGs) 33-35. To investigate whether the interaction of C-HOIL-1 with STAT1 attenuated STAT1 signaling, we examined STAT1 phosphorylation and ISG expressions upon IFN-β stimulation in R/K-GFP cells expressing wild-type or mutated C-HOIL-1. The up-regulated STAT1 phosphorylation over time in the R/K-GFP cells was significantly reduced by C-HOIL-1 and C295A (**Figure 4G**). The elevated expression of ISGs, including *CXCL9*, *CXCL10*, interferon regulatory factor 1 (*IRF1*), and guanine-binding protein 2 (*GBP2*), was suppressed by C-HOIL-1 and C295A (**Figure 4H-K**). These results demonstrated that C-HOIL-1 can attenuate STAT1 signaling and IFN-associated inflammatory responses; notably, such an action seems to be independent on the RBR function of C-HOIL-1.

### C-HOIL-1 inhibited M1-type macrophage polarization and inflammasome activation while up-regulating ARG1 expression

**Figure 5:**
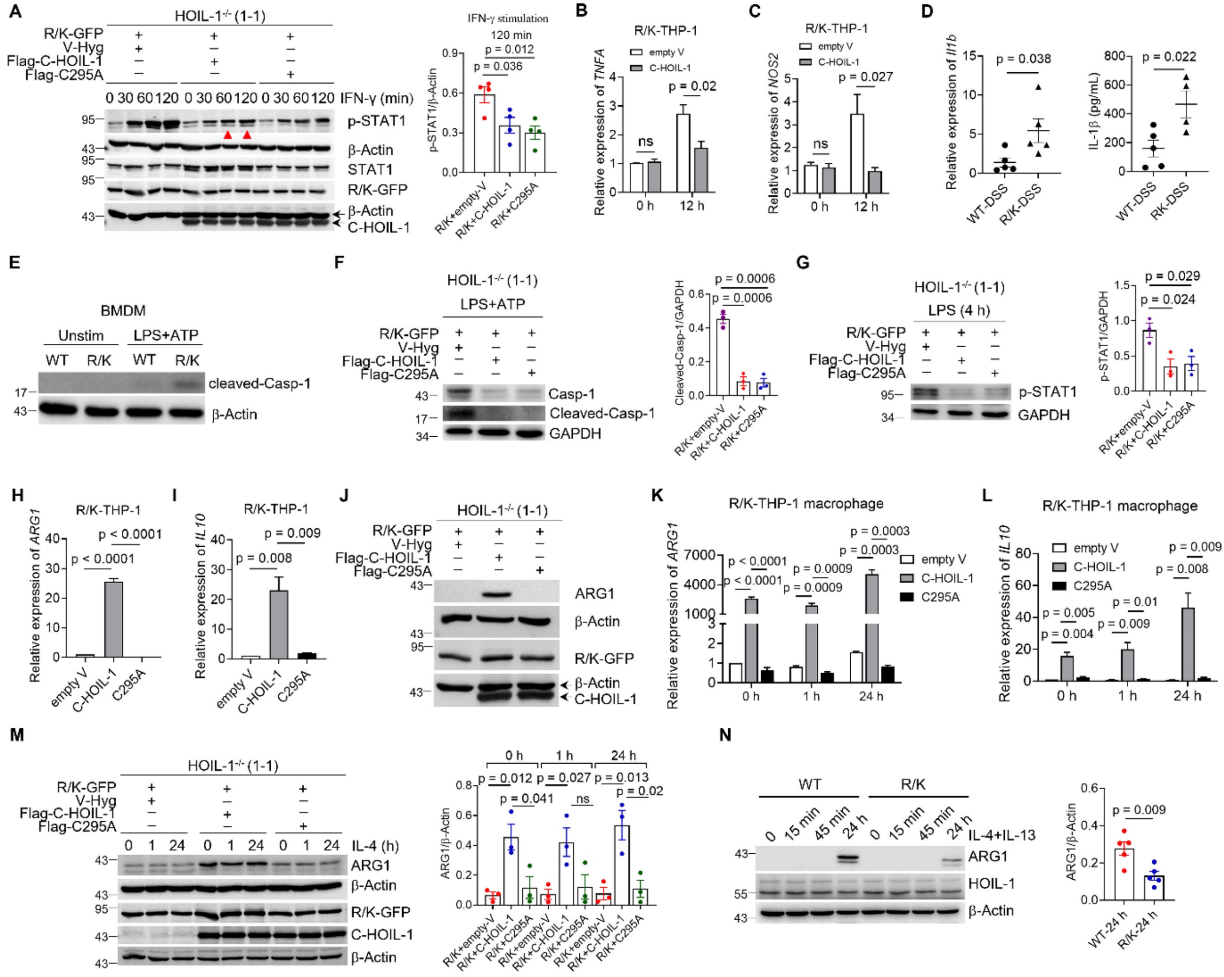
** Effects of C-HOIL-1 on STAT1-mediated inflammatory responses and ARG1 expression in monocytes and macrophages.** (**A**) Immunoblots showing the levels of STAT1 phosphorylation (p-STAT1) in R/K-GFP cells expressing V-Hyg, Flag-C-HOIL-1 or Flag-C295A upon IFN-γ (100 ng/mL) stimulation over time with quantitative analysis on p-STAT1 at 120 min on the right; β-Actin as the internal control. (**B, C**) The mRNA levels of *TNFA* (B) and *NOS2* (C) in R/K-GFP cell-derived macrophages expressing V-Hyg or Flag-C-HOIL-1 upon LPS (100 ng/mL) and IFN-γ (20 ng/mL) co-stimulation for 12 h by RT-qPCR; N = 4. (**D**) The expression of the inflammatory cytokine IL-1β gene (left) and protein (right) in the colon of WT and R/K mice with DSS-induce colitis by RT-qPCR and ELISA, respectively. (**E**) Immunoblots showing the cleaved-Casp-1 (activated form) in BMDM of WT and R/K mice with or without LPS (100 ng/mL, 4 h) and ATP (5 mM, 30 min) stimulation; β-Actin as the internal control. (**F**) Immunoblots showing the clevaged-Casp-1 in R/K-GFP cells expressing V-Hyg, Flag-C-HOIL-1, or Flag-C295A upon LPS (100 ng/mL, 4 h) and ATP (5 mM, 30 min) stimulation with quantitative analysis on the right; GAPDH as the internal control; N = 3. (**G**) Immunoblots showing the phosphorylation of STAT1 in R/K-GFP cells expressing V-Hyg, Flag-C-HOIL-1, or Flag-C295A with or without LPS (100 ng/mL, 4 h) stimulation with quantitative analysis on the right; GAPDH as the internal control; N = 3. (**H, I**) The relative expressions of *ARG1* (H) and *IL10* (I) in R/K-GFP cells expressing V-Hyg, Flag-C-HOIL-1, or Flag-C295A without stimulation by RT-qPCR; N = 3. (**J**) Immunoblots showing the protein expression of ARG1 in (H); β-Actin as the internal control. (**K, L**) The relative expressions of *ARG1* (K) and *IL10* (L) upon IL-4 (20 ng/mL) stimulation for 0, 1 and 24 h by RT-qPCR in R/K-GFP cell-derived macrophages expressing V-Hyg, Flag-C-HOIL-1, or Flag-C295A; N = 3. (**M**) Immunoblots showing the protein expression of ARG1 at the same condition in (K) with quantitative analysis on ARG1 expression at different time points on the right; β-Actin as the internal control. (**N**) Immunoblots showing the protein expression of ARG1 in BMDM of WT mice and R/K mice upon IL-4 (10 ng/mL) and IL-13 (10 ng/mL) co-stimulation over time with quantitative analysis at 24 h on the right; β-Actin as the internal control. ns: not significant.

Our *in vivo* results demonstrated that the percentage of inflammatory M1-type macrophages in the colon was elevated in the R/K mice during colitis (**Figure 2**). Since STAT1 activation is known to drive M1-type macrophage polarization 36-38, we hypothesized that such a process may be regulated by C-HOIL-1 through inhibiting STAT1 activation. We found that the expression of C-HOIL-1 and mutant C295A in the R/K-GFP cells suppressed STAT1 phosphorylation in response to IFN-γ stimulation (**Figure 5A**). Under the combined stimulation of LPS and IFN-γ for M1-type macrophage polarization, C-HOIL-1 also down-regulated the mRNA levels of M1-type signature genes *TNFA* and *NOS2* (**Figure 5B and C**). These results indicated that C-HOIL-1 is capable of inhibiting the IFN-γ-STAT1 signaling to hinder M1-type macrophage differentiation/polarization.

It is also known that STAT1 signaling can trigger classical NOD-, LRR- and pyrin domain-containing protein 3 (NLRP3)-mediated inflammasome activation for inflammatory responses in macrophages 39, 40. This activation is driven by a two-stage signaling cascade: (i) a priming signal initiated by the endotoxin (lipopolysaccharide, LPS) to up-regulate the expression of key proteins (e.g., NLRP3, pro-caspase-1, and pro-IL-1β), and (ii) an activation signal triggered by ATP (or damage associated molecular pattern, DAMP) for inflammasome assembly and inflammatory mediator release 41, 42. We first examined how the absence of C-HOIL-1 affects the inflammasome activation using the R/K mouse model of DSS-induced colitis. In the colon tissue of the R/K mice, both mRNA and protein levels of IL-1β were significantly higher than in the WT mice (**Figure 5D**). In addition, the levels of activated caspase-1 (cleaved-Casp-1) were strongly elevated in bone marrow derived macrophages (BMDMs) from R/K mice following NLRP3 inflammasome activation by LPS (4 h) and ATP (30 min) stimulation (**Figure 5E**). In R/K-GFP THP-1 cell-derived macrophages, the elevated levels of cleaved-Casp-1 were remarkably decreased by the expression of either C-HOIL-1 or C295A (**Figure 5F**). Since STAT1 activation primarily drives the priming stage of NLRP3 inflammasome formation, we investigated the effects of C-HOIL-1 on STAT1 activation by LPS stimulation (4 h) alone. We found that the LPS-induced phosphorylation of STAT1 in R/K-GFP cells was significantly inhibited by the presence of C-HOIL-1 or C295A (**Figure 5G**). These results revealed a novel regulatory function of C-HOIL-1 on STAT1 signaling to control NLRP3 inflammasome activation in macrophages.

To our curiosity, the effect of C-HOIL-1 on the anti-inflammatory signaling in monocytes/macrophages was also investigated. Surprisingly, the expression of typical anti-inflammatory genes *ARG1* and* IL10* was dramatically up-regulated by C-HOIL-1, but not by C295A in the R/K cells at the resting stage (**Figure 5H-J**). The same phenomenon was observed when these cells were differentiated into macrophages with or without IL-4 (20 ng/mL) stimulation at various time points (**Figure 5K-M**). In the BMDMs from the R/K and WT mice, the induction of ARG1 expression following co-stimulation with mIL-4 (10 ng/mL) and mIL-13 (10 ng/mL) for 24 h was significantly reduced in the R/K group compared with the WT group (**Figure 5N**), indicating that the absence of C-HOIL-1 compromises the induction of ARG1 expression in mouse BMDMs.

Collectively, we identified the dual molecular functions of C-HOIL-1 in regulating macrophage polarization and inflammatory responsiveness. Specifically, C-HOIL-1 (i) binds to STAT1 and inhibits STAT1 signal transduction, thereby reducing M1-type gene expression and suppressing NLRP3 inflammasome activation, and (ii) up-regulates the expression of the anti-inflammatory genes *ARG1* and *IL10*. Through this combined mechanism, C-HOIL-1 could serve as a critical regulator of M1-type macrophage differentiation/polarization and the overall inflammatory responses.

### The myeloid-specific HOIL-1 cleavage was essential for controlling intestinal inflammation in the DSS-induced colitis mouse model

Based on our findings so far, to further verify whether myeloid cells (e.g., monocytes and macrophages) drive the exacerbated DSS-induced colitis observed in the R/K mice, we constructed the myeloid-specific uncleavable HOIL-1 mouse model (R/K^flox/flox^Lyz2-Cre^+^). This was achieved by crossing a gene-edited conditional R/K point mutation line (R/K^flox/flox^) with Lyz2-Cre^+^ mice (**Figure S13**). The homologous R/K^flox/flox^Lyz2-Cre^+^ mice (i.e., the myeloid-specific R/K mice) were compared with the Cre^-^ control littermates (R/K^flox/flox^Lyz2-Cre^-^).

**Figure 6:**
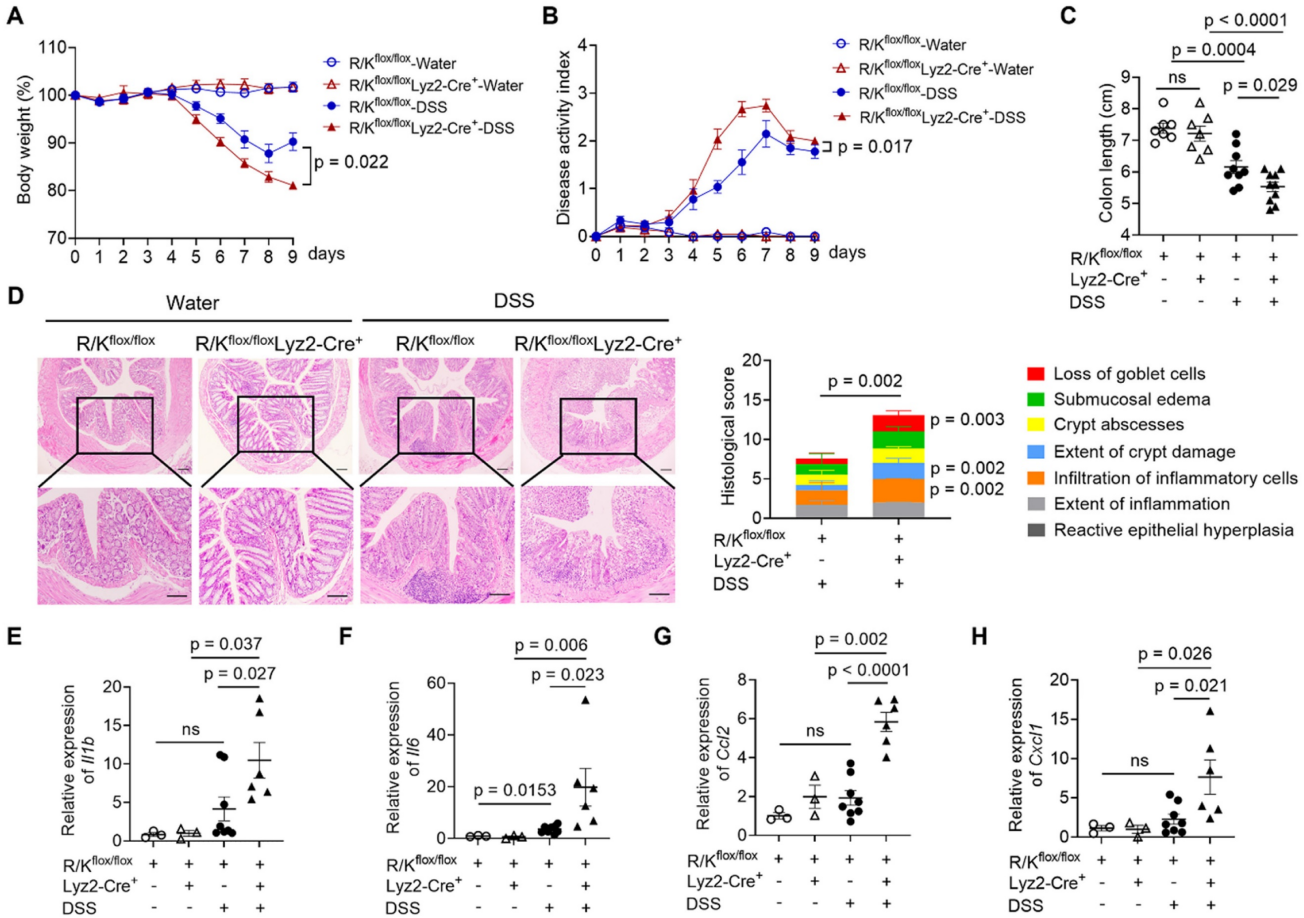
** Myeloid cell-specific abnormality of HOIL-1 cleavage aggravates DSS-induced colitis in mice.** (**A-D**) The body weight loss (**A**), the disease activity index (DAI) (**B**), the colon length (**C**), and the H&E staining (left) with the histological scores (right) (**D**) of R/K^flox/flox^ and R/K^flox/flox^Lyz2-Cre^+^ mice with or without DSS (2.5%)-induced colitis; scale bar = 100 µm; N = 7 for water groups, N = 9 for DSS groups. (**E-H**) The relative expression of the inflammatory genes *Il1b* (**E**), *Il6* (**F**), *Ccl2* (**G**), and *Cxcl1* (**H**) in the colon tissues of R/K^flox/flox^ and R/K^flox/flox^Lyz2-Cre^+^ mice with or without DSS-induced colitis measured by RT-qPCR; N = 3 for water groups, N = 8 for WT-DSS group, N = 6 for R/K-DSS group. ns: not significant.

The same DSS-induced colitis model was employed to evaluate the impact of myeloid-specific HOIL-1 cleavage on the colonic inflammation. Similar to the global R/K mutants, myeloid-specific R/K mice displayed increased susceptibility to colitis, evidenced by a larger weight loss (**Figure 6A**), higher DAI scores (**Figure 6B**), and a shorter colon length (**Figure 6C**). The histological analysis confirmed that R/K^flox/flox^Lyz2-Cre^+^ mice had worse colonic inflammation and damage, particularly on the loss of goblet cells, extent of crypt damage, and infiltration of inflammatory cells (**Figure 6D**) than the controls. Additionally, the expression of key inflammatory genes, such as *Il1b*, *Il6*, *Ccl2*, and *Cxcl1*, was significantly elevated in the colon tissues of myeloid-specific R/K mice in comparison with the controls (**Figure 6E-H**). These results confirmed that myeloid-specific HOIL-1 cleavage is critical to regulate the intestinal inflammation.

### Expressing C-HOIL-1 in the colon alleviated DSS-induced colitis in mice

**Figure 7:**
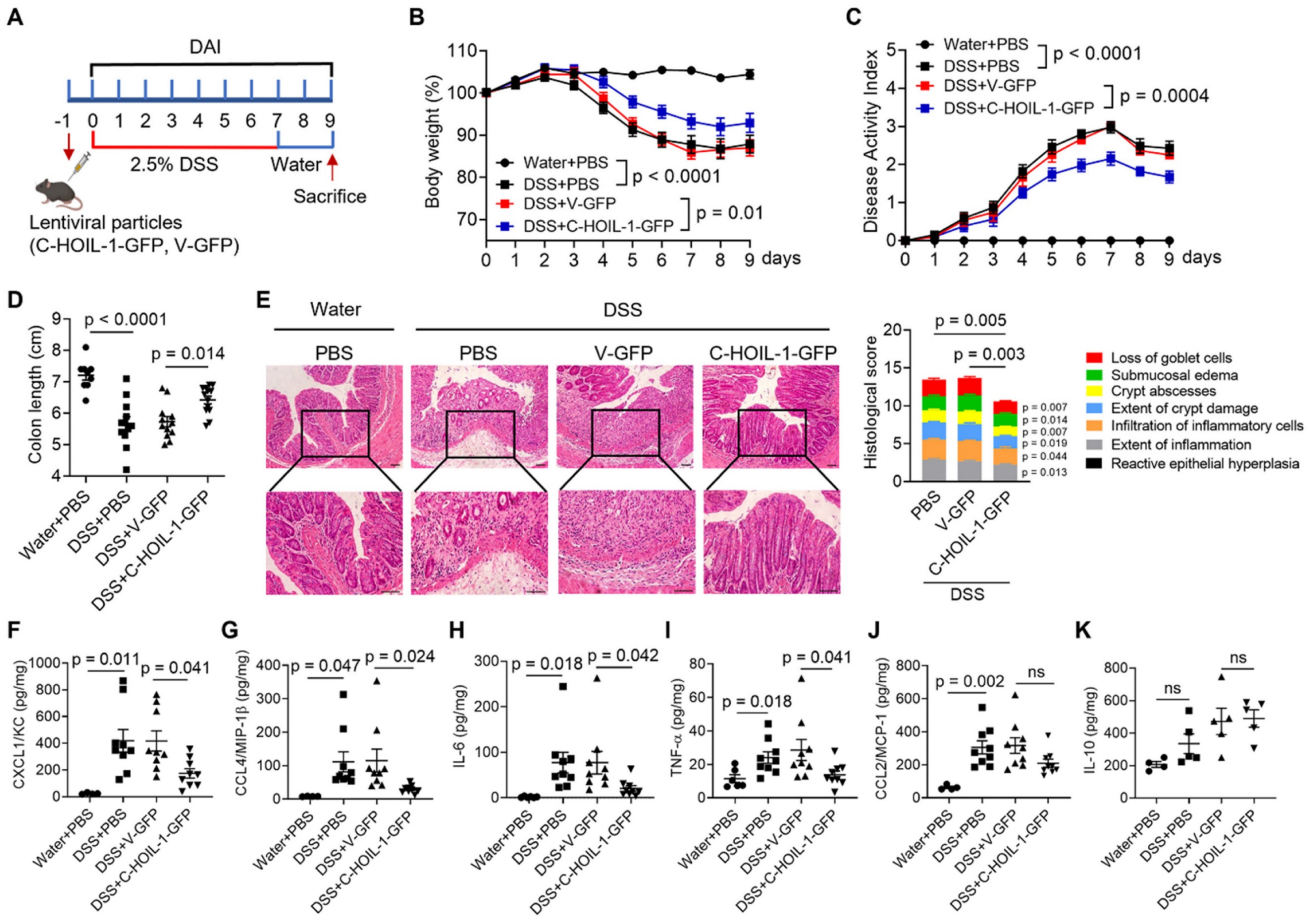
** Pretreatment of C-HOIL-1 lentiviral particles via enema alleviates DSS-induced colitis in mice.** (**A**) A schematic diagram illustrating the pretreatment (on Day -1) of C-HOIL-1 lentiviral particles (C-HOIL-1-GFP) or the empty vector (V-GFP) control by enema in WT mice with DSS (2.5%)-induced colitis. (**B-D**) The effects of the pretreatment of C-HOIL-1 lentiviral particles on the mouse body weight loss (**B**), the DAI scores (**C**), and the colon length (**D**) under DSS-induced colitis in comparison with V-GFP; N = 9 for water groups, N = 13 for DSS groups. (**E**) The H&E images of the colon tissues (left) and their histological scores (right) showing the reduced colon inflammation and injury by C-HOIL-1-GFP pretreatment compared with the V-GFP group; scale bar = 100 µm; N = 8. (**F-K**) The production of cytokines CXCL1/KC (**F**), CCL4/MIP-1β (**G**), IL-6 (**H**), TNF-α (**I**), CCL2/MCP-1 (**J**) and IL-10 (**K**) in the colon tissues of mice with the pretreatment of C-HOIL-1-GFP or V-GFP under DSS-induced colitis; N = 4 for water groups, N = 9 for DSS groups in (F-J); N = 4 for water groups, N = 5 for DSS groups in (K). ns: not significant.

In order to validate the regulatory function and therapeutic potential of C-HOIL-1 in colitis, the WT mice were pretreated with Lenti-C-HOIL-1 particles via enema 1 day prior to 2.5% DSS induction (**Figure 7A**). While the weight loss profiles and DAI scores confirmed the successful establishment of DSS-induced colitis, these symptoms were alleviated by the Lenti-C-HOIL-1 pretreatment (C-HOIL-1-GFP) when compared with the empty Lenti-vector control (V-GFP) (**Figure 7B and C**). The C-HOIL-1 pretreatment also prevented the shortening of the colon length (**Figure S14A, Figure 7D**). The histological analysis confirmed that the C-HOIL-1-GFP groups exhibited reduced colonic inflammation and tissue injury in comparison with the PBS or V-GFP treated mice (**Figure 7E**). Moreover, the Lenti-C-HOIL-1 pretreatment significantly down-regulated the production of several pro-inflammatory cytokines CXCL1/KC, CCL4/MIP-1β, IL-6 and TNF-α in the colon (**Figure 7F-I**). However, no significant effects were observed on CCL2/MCP-1 (**Figure 7J**) or IL-10 (**Figure 7K**) production when compared with the V-GFP group.

**Figure 8:**
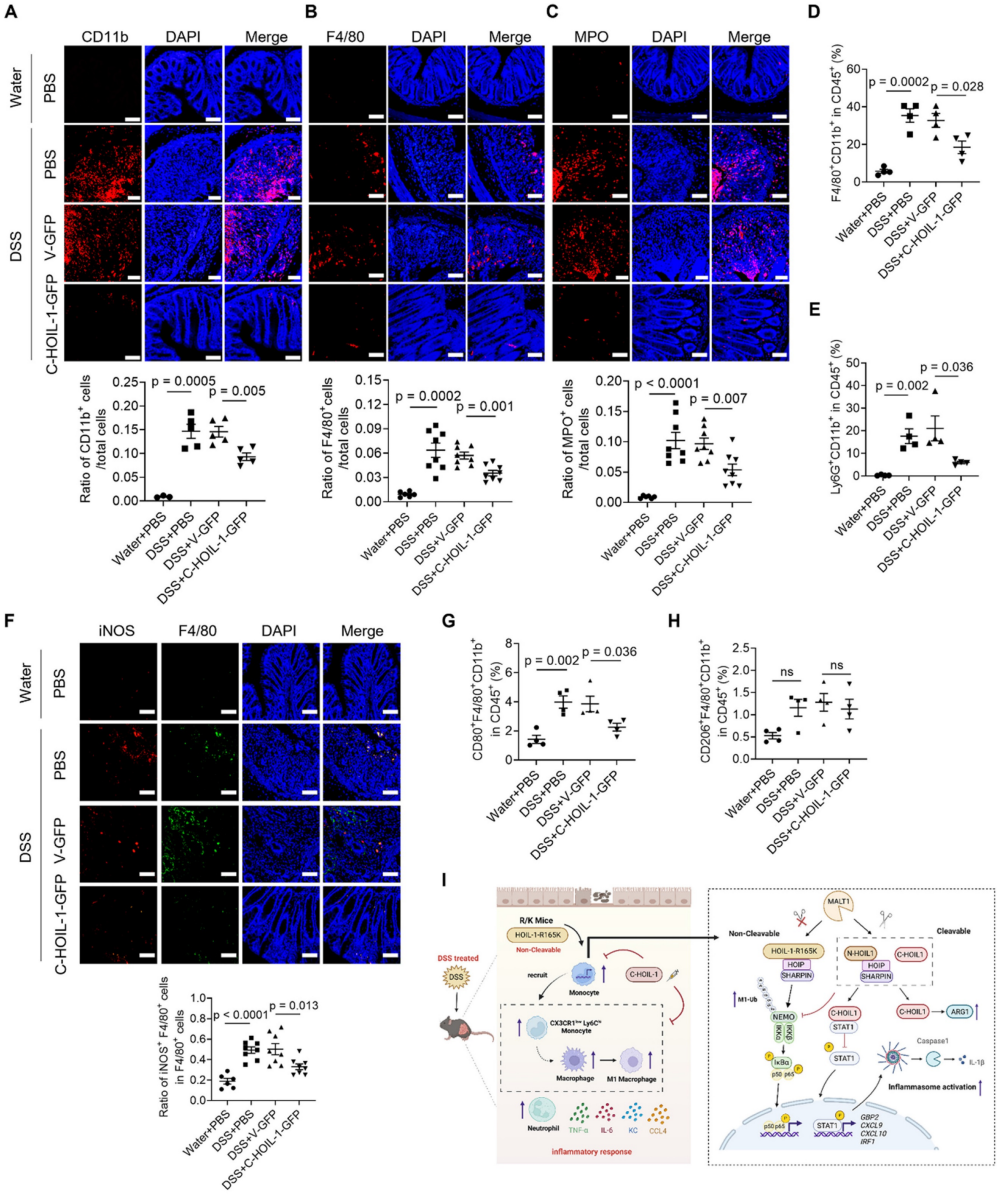
** The pretreatment of C-HOIL-1 lentiviral particles reduces the infiltration of inflammatory myeloid cells to the colon in colitis mice.** (**A-C**) Immunofluorescence images showing the infiltrated CD11b^+^ myeloid cells (**A**), F4/80^+^ macrophages (**B**), and MPO^+^ neutrophils (**C**) in the colon of the control mice and colitis mice with or without the C-HOIL-1-GFP pretreatment; V-GFP as the viral control; the quantitative analysis on the cell percentage shown at the bottom; scale bar = 100 µm; N = 3 for water groups, N = 5 for DSS groups in (A); N = 6 for water groups, N = 8 for DSS groups in (B, C). (**D, E**) The percentage of F4/80^+^CD11b^+^ macrophages (**D**) and Ly6G^+^CD11b^+^ neutrophils (**E**) in the colonic lamina propria of the control mice and colitis mice with or without the pretreatment of C-HOIL-1-GFP and V-GFP by flow cytometry analysis; N = 4. (**F**) Immunofluorescence images showing the percentage of F4/80^+^iNOS^+^ M1-type macrophages in the colon of the control mice and colitis mice with or without the pretreatment of C-HOIL-1-GFP and V-GFP with quantitative analysis at the bottom; scale bar = 100 µm; N = 6 for water groups, N = 8 for DSS groups. (**G, H**) The percentage of F4/80^+^CD11b^+^CD80^+^ M1-type macrophages (**G**) and F4/80^+^CD11b^+^CD206^+^ M2-type macrophages (**H**) in the colonic lamina propria of the control mice and colitis mice with or without the pretreatment of C-HOIL-1-GFP and V-GFP by flow cytometry analysis; N = 4. (**I**) A working mechanism of HOIL-1 cleavage and the C-HOIL-1 fragment in regulating DSS-induced intestinal inflammation was proposed. The defect in HOIL-1 cleavage aggravates DSS-induced colitis by increasing the infiltration of inflammatory monocytes, M1-type macrophages, and neutrophils in the colon, which is reversed by the C-HOIL-1 lentiviral particles pretreatment directly to the colon. At the molecular level, the abnormality of HOIL-1 cleavage increases NF-κB activation and inflammatory responses in monocytes, while the presence of C-HOIL-1 reverses such reaction. Mechanistically, C-HOIL-1 can bind to STAT1 to inhibit the downstream signaling and classical inflammasome activation; on the other hand, C-HOIL-1 can also up-regulate the expression of ARG1. These actions together hinder the inflammatory responses and differentiation of M1-type macrophages, contributing to the reduction in colon inflammation; the graph was created with BioRender.com. ns: not significant.

We next examined the effects of Lenti-C-HOIL-1 pretreatment on the infiltration of inflammatory immune cells to the colon. First, the immunofluorescence staining revealed that the Lenti-C-HOIL-1 treatment significantly reduced the infiltration of the myeloid cells (CD11b^+^), macrophages (F4/80^+^), and neutrophils (MPO^+^) compared to the V-GFP controls (**Figure 8A-C**); however, the infiltration of CD4^+^ T cells was not affected (**Figure S14B**). These results were confirmed by the flow cytometry analysis on the immune cell population in the colonic lamina propria. It was found that the elevated percentage of F4/80^+^CD11b^+^ macrophages and Ly6G^+^CD11b^+^ neutrophils by DSS induction was significantly reduced by the Lenti-C-HOIL-1 pretreatment (**Figure 8D and E**). Second, the percentage of iNOS^+^ (by immunofluorescence) or F4/80^+^CD80^+^CD11b^+^ (by flow cytometry analysis) M1-type macrophages in the colon of colitis mice was significantly decreased by Lenti-C-HOIL-1 (**Figure 8F and G**). Notably, we did not observe significant effects of Lenti-C-HOIL-1 on the percentage of CD163^+^ (**Figure S14C**) or F4/80^+^CD206^+^CD11b^+^ (**Figure 8H**) M2-type macrophages. These results confirmed that expression of C-HOIL-1 in the colon alleviated DSS-induced colitis by suppressing inflammatory myeloid cell infiltration.

Based on all these findings, a hypothetical working model was proposed (**Figure 8I**). In this model, the defect in HOIL-1 cleavage aggravates DSS-induced colitis by promoting the infiltration of inflammatory monocytes, M1-type macrophages, and neutrophils to the colon. The delivery of Lenti-C-HOIL-1 directly to the colon reverses this pathology. Mechanistically, the resulting C-HOIL-1 from HOIL-1 cleavage functions as a dual regulator: it inhibits NF-κB signaling and binds to STAT1 to suppress downstream ISG expressions and NLRP3 inflammasome activation, while simultaneously up-regulating ARG1 expression. These molecular events together constrain myeloid inflammatory responses and M1-type macrophage differentiation/polarization, leading to the reduction of colonic inflammation.

## Discussion

We and others previously discovered that HOIL-1 cleavage by MALT1 negatively regulates NF-κB signaling through destructing LUBAC stability and catalytic function of linear ubiquitination 13-15. By establishing uncleavable HOIL-1 (HOIL-1-R165K) in HOIL-1 deficient human fibroblasts, we further demonstrated that inability to cleave HOIL-1 exacerbates TNF-α and IL-1β-mediated inflammatory responses 16. In order to define the mechanisms of HOIL-1 cleavage and its released C-HOIL-1 fragment in regulating intestinal inflammation, in this study, we constructed a transgenic mouse model of HOIL-1-R/K (lack of C-HOIL-1) and genetically engineered THP-1 monocytic cells. The *in vivo* work revealed that R/K mice exhibit a deteriorate disease phenotype in DSS-induced colitis when compared with the WT mice. The exacerbated intestinal inflammation is characterized by the significantly increased infiltration of inflammatory Ly6C^hi^CX3CR1^low^CD11b^+^ monocytes, M1-type macrophages, and neutrophils in the colon. In THP-1 cells, we confirmed that the enhanced TNF-α-mediated NF-κB activation and inflammatory responses in the HOIL-1-R/K condition can be reversed by the restoration of C-HOIL-1. Mechanistically, C-HOIL-1 can directly interact with STAT1 and inhibit STAT1 signaling, which in turn suppresses NLRP3 inflammasome activation and M1-type macrophage polarization. These myeloid-cell specific hyper-inflammatory responses were further validated using the conditional HOIL-1-R/K knock-in mice (R/K^flox/flox^Lyz2-Cre^+^). To explore the potential application of C-HOIL-1 as a therapeutic agent in controlling gut inflammation, we delivered Lenti-C-HOIL-1 to the colon of WT mice by enema, and significantly alleviated DSS-induced colitis. Our work disclosed novel functions of C-HOIL-1 in regulating inflammatory responses of monocytes and macrophages, and provided a new strategy to manage intestinal inflammation of IBD.

### HOIL-1 cleavage regulates NF-κB signaling pathway and inflammatory responses of myeloid cells to suppress gut inflammation

Excessive activation of the NF-κB signaling pathway has been found to participate in the pathogenesis of IBD 2, 3. In IBD patients, NF-κB is highly up-regulated in mucosal cells, macrophages, and intestinal epithelial cells 43, 44. In addition, pharmacological inhibition of this pathway has proven effective in reducing colitis in murine models 43. Moreover, several IBD genetic risk alleles, such as *NOD2*, *TOLLIP*, and *TNFIAP3* (encoding the deubiquitinase A20), contribute to disease progression through the dysregulation of NF-κB signaling 45. These facts highlight the pivotal role of dysregulated NF-κB pathway in gut inflammation.

We previously identified that MALT1 cleaves HOIL-1 to down-regulate NF-κB signaling 13, which provides a reasonable explanation for the IBD-like phenotype observed in MALT1 deficiency. Using a stable transduction system to express uncleavable HOIL-1 and WT HOIL-1 in HOIL-1 deficient patient skin fibroblasts, we further confirmed that impaired HOIL-1 cleavage promotes heightened NF-κB activation and inflammatory responses 16. Consistent with these findings, the present study demonstrates that the defect in HOIL-1 cleavage enhances TNF-α-mediated NF-κB activation and pro-inflammatory cytokine production in R/K THP-1 monocytes (**Figure 3C-E**), underscoring the universal regulatory role of HOIL-1 cleavage in this pathway. By using the established R/K mice, the defect in HOIL-1 cleavage indeed promotes a more severe colonic inflammatory phenotype in a DSS-induced colitis model when compared with the WT controls (**Figure 1**). This severity was found to be associated with the increased infiltration of inflammatory Ly6C^hi^CX3CR1^low^CD11b^+^ circulating monocytes, M1-type macrophages, and neutrophils into the colon (**Figure 2**). Notably, the time-dependent immune profiling (**Figure 2D-I**) suggests that the circulating inflammatory monocytes may act as the initiating factor to facilitate subsequent M1-type macrophage differentiation and neutrophil recruitment 46, 47. These findings confirmed that defective HOIL-1 cleavage drives the excessive inflammatory responses in myeloid cells and exacerbates DSS-induced colitis.

It should be noted that I. Skordos *et al*. recently reported a MALT1 protease-resistant HOIL-1 knock-in mouse model (HOIL-1-R165A) 48. Interestingly, this mutant HOIL-1 does not affect the development of CD4^+^T cells, CD8^+^T cells, and Treg cells in the thymus as well as B cells in the spleen. They also found that B cells and CD4^+^T cells have normal responses in NF-κB and MAPK pathways upon PKC-mediated stimulation. These findings support our results of R/K mice, where the colonic infiltration of CD4^+^T cells was not affected during DSS-induced colitis (**Figure S3**). In fact, by using the myeloid cell-specific R/K mice, we confirmed the importance of myeloid-cell specific HOIL-1 cleavage in the regulation of intestinal inflammation (**Figure 6**).

### C-HOIL-1 has new biological functions in suppressing inflammatory responses of monocytes and macrophages

HOIL-1 cleavage produces two distinct fragments N-HOIL-1 and C-HOIL-1. While N-HOIL-1 retains the ability to interact with HOIP and SHARPIN through its UBL domain (**Figure 3F, Figure S9B**), C-HOIL-1 is released from LUBAC. Several lines of evidence suggest that C-HOIL-1 may possess novel biological functions: (i) C-HOIL-1 persists in the cells for up to 24 h 13; (ii) it bears the RBR E3 ligase domain; (iii) it has a distinct intracellular localization pattern different from the full-length HOIL-1-R/K (**Figure 3K**); (iv) while HOIL-1 deficiency is associated with autoinflammation 9, 10, the defect in HOIL-1 cleavage also promotes inflammatory responses 16. Confirming this hypothesis, we indeed observed that C-HOIL-1 is capable of inhibiting TNF-α-induced NF-κB activation and cytokine production (**Figure 3G-J**). Mechanistically, we identified that C-HOIL-1 directly binds to STAT1 (**Figure 4B-F, Figures S11 and S12**), down-regulates IFN-induced ISG expression (**Figure 4G-K**), reduces the expression of M1-type macrophage signature genes (**Figure 5B and C**), and inhibits NLRP3 inflammasome activation (**Figure 5D-G**). Moreover, C-HOIL-1 significantly up-regulates *ARG1* and *IL10* expression in THP-1 monocytes and macrophages with/without stimulation (**Figure 5H-M**). These results uncover a novel molecular function of C-HOIL-1 in inhibiting STAT1 signaling and associated inflammatory responses in monocytes and macrophages.

Although, we discovered that C-HOIL-1 binds to STAT1 and inhibits its phosphorylation upon IFN stimulation (**Figure 4D-G and Figure 5A**), the precise molecular mechanism of how C-HOIL-1 regulates STAT1 phosphorylation remains to be elucidated. Structurally, C-HOIL-1 consists of a NZF domain proximal to the cleavage site and a RBR domain at the C-terminal (**Figure 3F**). The NZF domain is known to selectively bind to linear ubiquitin chains 49, whereas the RBR domain catalyzes the E3 ligase activity 50. The presence of these domains in C-HOIL-1 suggests two possible mechanisms by which C-HOIL-1 inhibits STAT1 activation. First, the NZF domain may facilitate the binding of linear ubiquitin chains to STAT1, impeding the interaction of STAT1 with IFNAR2 for subsequent phosphorylation 51. Second, the RBR domain might catalyze STAT1 ubiquitination, leading to its proteasomal degradation. Future investigations are required to distinguish between these possibilities.

In addition to stabilizing LUBAC structure, HOIL-1 can also function as an E3 ligase through the C-terminal RBR domain 52. It has been found that HOIL-1 catalyzes the K48-linked polyubiquitination on iron oxide regulatory protein 2 (IRP2) 53 and PKCζ 54 to facilitate their proteasomal degradation. More recently, a new function of HOIL-1 E3 activity was identified to catalyze the formation of oxyester bonds between the ubiquitin and substrates, resulting in mono-ubiquitination 27. The addition of mono-ubiquitin on LUBAC by HOIL-1 results in reduced LUBAC-mediated linear ubiquitination on target substrates, thereby decreasing LUBAC associated inflammatory signaling 28, 31. These diverse E3 ligase activities of HOIL-1 suggest that the RBR domain within C-HOIL-1 may contribute to its functionality. Unexpectedly, by comparing wild-type C-HOIL-1 with its enzymatic mutant (C295A), the observed C-HOIL-1-mediated inhibition on total linear and K48-linked ubiquitination (**Figure 4A**) as well as TNF-α-induced NF-κB activation was not reversed by the C295A mutation (**Figure S10A**). These findings indicate that the RBR function of C-HOIL-1 might not be involved in regulating NF-κB-mediated inflammatory responses in this context.

Interestingly, we observed a significant difference in ARG1 expression between cells expressing wild-type C-HOIL-1 and those expressing catalytically inactive C-HOIL-1 (C295A) mutant (**Figure 5H-M**). As ARG1 expression is regulated by several transcription factors, including STAT3, STAT6, PPARγ, and CREB 55, the E3 activity of C-HOIL-1 may modulate one or more of these pathways. Further unbiased analysis and targeted investigations are required to identify the specific signaling pathway through which C-HOIL-1 enzymatically drives *ARG1* up-regulation.

### C-HOIL-1 as a potential therapeutic agent for treating IBD

Sustained uncontrolled intestinal inflammation is the pathological hallmark of IBD that lacks curable therapy at the moment. The multi-factorial etiology of IBD development and progression makes the search for effective pharmacological therapeutics particularly challenging. Current management of gut inflammation in IBD patients primarily relies on conventional corticosteroids, small molecule immunomodulators (e.g., sphingosine-1-phosphate receptor modulator), and emerging biologics such as infliximab (anti-TNF-α), ustekinumab (anti-IL-12/IL-23), and vedolizumab (anti-integrin α4β7) 56, 57. However, these systemic therapies are often associated with adverse side effects and limited long-term efficacy due to the disease heterogeneity; up to 30% of patients would develop drug resistance after long-term use of these biologics 58. Therefore, there is an urgent need to discover a more specific and effective therapeutic strategy to treat IBD.

In this study, we defined the critical role of HOIL-1 cleavage and its product C-HOIL-1 in the regulation of intestinal inflammation. By using transgenic mice, we demonstrated that lack of C-HOIL-1 (uncleavable HOIL-1) promotes the infiltration of inflammatory myeloid cells into the colon and exacerbates DSS-induced colitis (**Figures 1 and 2**); these pathogenic characteristics appeared to be myeloid-specific (**Figure 6**). When the C-HOIL-1 lentiviral particles were given to the WT mice via enema prior to DSS challenge, the disease phenotypes and intestinal inflammation were significantly alleviated (**Figures 7 and 8**). These results suggest that targeted delivery of C-HOIL-1 to intestinal monocytes and macrophages represents a promising therapeutic strategy to ameliorate colitis. Although this study provides a proof-of-principle for C-HOIL-1 as a potential treatment for gut inflammation, the optimal administration routes and the delivery approach warrant further investigation. For example, a proper designed delivery nano-vehicle may be required for oral administration to achieve targeted C-HOIL-1 expression in the inflammatory monocytes and macrophages in the gut. Overall, our studies define the functionality of C-HOIL-1 released from HOIL-1 cleavage, and offer a novel approach for controlling intestinal inflammation and potentially other diseases characterized by myeloid-driven inflammation.

## Conclusions

In this study, we defined the role of HOIL-1 cleavage and discovered novel functions of C-HOIL-1 in regulating intestinal inflammatory responses. By using a transgenic mouse model expressing uncleavable HOIL-1 (HOIL-1-R165K; R/K), we demonstrated that R/K mice exhibit an exacerbated disease phenotype in DSS-induced colitis with increased colonic infiltration of inflammatory Ly6C^hi^CX3CR1^low^CD11b^+^ monocytes, M1-type macrophages, and neutrophils. The defect in HOIL-1 cleavage also promoted TNF-α-induced NF-κB activation and inflammatory responses in THP-1 monocytes, and elevated the production of inflammatory mediator IL-1β in the colon. Mechanistically, we identified the molecular functions of C-HOIL-1 in direct interaction with STAT1 to down-regulate ISG expression; more importantly, STAT1 inhibition by C-HOIL-1 decreased M1-type signature gene expression and strongly suppressed NLRP3 inflammasome activation in macrophages. In addition, C-HOIL-1 up-regulated *ARG1* expression drastically. These actions ultimately suppressed the inflammatory responses and M1-type polarization in macrophages. Moreover, the importance of myeloid-specific HOIL-1 cleavage in regulating gut inflammation was confirmed using a conditional knock-in HOIL-1-R/K (R/K^flox/flox^Lyz2-Cre^+^) mouse model. Finally, the administration of C-HOIL-1 lentiviral particles by enema significantly alleviated colitis in WT mice with decreased colonic infiltration of inflammatory myeloid cells. Our work for the first time uncovers the biological mystery of the HOIL-1 cleavage product C-HOIL-1 in regulating myeloid inflammatory responses, offering new strategies to manage excessive intestinal inflammation in IBD patients.

## Supplementary Material

Supplementary figures describing the abnormality of HOIL-1 cleavage promoting NF-κB activation in splenic mononuclear cells of R/K mice, IL-17A and IL-5 levels and CD4^+^T cell infiltration in the colon of R/K colitis mice, multicolor flow analysis on different myeloid cell populations and infiltration of different sub-types of macrophages in the colon, construction and validation of HOIL-1^-/-^ THP-1 cells, MALT1-dependent cleavage of HOIL-1 in THP-1 cells, negative regulation of NF-κB signaling by HOIL-1 cleavage, the inability of C-HOIL-1 in interacting and stabilizing HOIP and SHARPIN, effects of mutant C-HOIL-1 on NF-κB activation and IL-8 production, validation of the C-HOIL-1 interacting proteins, physical interaction of C-HOIL-1 with STAT1, genotype identification of myeloid cell-specific R/K mice, and effects of C-HOIL-1 lentiviral particle pretreatment on the colon length and immune cell infiltration in WT colitis mice as well as supplementary tables listing primer sequences, criteria for DAI and histological scoring, antibodies information, and sgRNA sequences.

## Data Availability

All data generated and analyzed during this research are included in this article and available upon request.
