# Supplementary material for: C-terminal Fragment Generated by HOIL-1 Cleavage Suppresses Inflammatory Responses of Myeloid Cells to Alleviate Colitis
Source: Theranostics. 2026 Feb 11;16(9):4580–602. doi: 10.7150/thno.124294 (PMC12964130; doi:10.7150/thno.124294)
Supplement: Supplementary file 1 — Supplementary figures describing the abnormality of HOIL-1 cleavage promoting NF-κB activation in splenic mononuclear cells of R/K mice, IL-17A and IL-5 levels and CD4+T cell infiltration in the colon of R/K colitis mice, multicolor flow analysis on different myeloid cell populations and infiltration of different sub-types of macrophages in the colon, construction and validation of HOIL-1-/- THP-1 cells, MALT1-dependent cleavage of HOIL-1 in THP-1 cells, negative regulation of NF-κB signaling by HOIL-1 cleavage, the inability of C-HOIL-1 in interacting and stabilizing HOIP and SHARPIN, effects of mutant C-HOIL-1 on NF-κB activation and IL-8 production, validation of the C-HOIL-1 interacting proteins, physical interaction of C-HOIL-1 with STAT1, genotype identification of myeloid cell-specific R/K mice, and effects of C-HOIL-1 lentiviral particle pretreatment on the colon length and immune cell infiltration in WT colitis mice as well as supplementary tables listing primer sequences, criteria for DAI and histological scoring, antibodies information, and sgRNA sequences. [file thnov16p4580s1.pdf]

Supplementary Material

C-terminal fragment generated by HOIL-1 cleavage suppresses inflammatory responses of myeloid cells to alleviate colitis

Xiaomeng Li[1,#], Hefan Zhang[1,#], Qian Wang[1,#], Qianqian Li[1,#], Xingru Wang[1], Yu Tian[1], Rui Zhang[1], Qiuyun Chen[1], Christopher M. Overall[2], Stuart E. Turvey[3], Bangmao Wang[4], Hailong Cao[4], Hong Yang[5,], Shan-Yu Fung[1,]**

[1] State Key Laboratory of Experimental Hematology, Department of Immunology and Key Laboratory of Immune Microenvironment and Disease (Ministry of Education), School of Basic Medical Science, The Province and Ministry Co-Sponsored Collaborative Innovation Center for Medical Epigenetics, International Joint Laboratory of Ocular Diseases, Ministry of Education, Tianjin Medical University, Tianjin, China

[2] Department of Biochemistry and Molecular Biology, Department of Oral Biological and Medical Science, Center for Blood Research, The University of British Columbia, Vancouver, Canada

[3] Department of Pediatrics, British Columbia Children's Hospital, Experimental Medicine Program, Faculty of Medicine, University of British Columbia, Vancouver, Canada

[4] Department of Gastroenterology and Hepatology, General Hospital, Tianjin Medical University, Tianjin Institute of Digestive Diseases, Tianjin Key Laboratory of Digestive Diseases, Tianjin, China

[5] Department of Pharmacology and Tianjin Key Laboratory of Inflammatory Biology, School of Basic Medical Sciences, The Province and Ministry Co-Sponsored Collaborative Innovation Center for Medical Epigenetics, International Joint Laboratory of Ocular Diseases, Ministry of Education, Intensive Care Unit of the Second Hospital, Tianjin Medical University, Tianjin, China

#These authors contributed equally to this paper.

*Corresponding authors: Professor Shan-Yu Fung (E-mail: shanefung@tmu.edu.cn); Professor Hong Yang (E-mail: hongyang@tmu.edu.cn)

Keywords: inflammatory bowel disease, macrophage, HOIL-1 cleavage, STAT1, immunotherapy

List of contents

Supplementary methods

Extraction and stimulation of splenic mononuclear cells

Culture of mouse bone marrow-derived macrophages

Preparation of purified recombinant C-HOIL-1 and STAT1 proteins

Supplementary figures

Figure S1 The abnormality of HOIL-1 cleavage promoted NF-κB activation in splenic mononuclear cells from the R/K mice.

Figure S2 Production of IL-17A and IL-5 cytokines in the WT and R/K mice under DSS-induced colitis.

Figure S3 The infiltration of $CD4^{+}$T cells in the colon of WT and R/K mice under DSS-induced colitis.

Figure S4 The multicolor flow analysis identifying different myeloid cell populations in the colonic lamina propria.

Figure S5 The infiltration of different sub-types of macrophages in the colon.

Figure S6 Construction and validation of HOIL-$1^{-/-}$ THP-1 cells by CRISPR/Cas9 approach.

Figure S7 MALT1-dependent cleavage of HOIL-1 in THP-1 cells.

Figure S8 Negative regulation of NF-κB signaling by HOIL-1 cleavage.

Figure S9 The inability of C-HOIL-1 in interacting and stabilizing HOIP and SHARPIN proteins.

Figure S10 The effects of mutant C-HOIL-1 on NF-κB activation and IL-8 production.

Figure S11 Validation of the C-HOIL-1 interacting proteins identified by IP-MS assay.

Figure S12 The direct physical interaction between C-HOIL1 and STAT1 in vitro.

Figure S13 Genotype identification of mice with myeloid cell-specific R/K mutation.

Figure S14 Effects of the pretreatment of C-HOIL-1 lentiviral particles on colon length and immune cell infiltration in the WT mice under DSS-induced colitis.

Supplementary tables

Table S1 List of primer sequences used in this study.

Table S2 Criteria for DAI scoring.

Table S3 Criteria for the histological assessment and scoring.

Table S4 List of antibodies information in this study.

Table S5 SgRNA sequences.

Supplementary methods

Extraction and stimulation of splenic mononuclear cells

WT and R/K mice aged 8 to 12 weeks were sacrificed. Spleens were harvested and carefully minced. These spleen tissues were then placed on a 70-μm nylon mesh and gently ground with a 1-mL syringe, followed by rinsing with PBS solution. The cell suspension was centrifuged, and processed with a red blood cell lysis buffer. Next, the resuspended cells were layered on the top surface of Ficoll-Paque separation solution (Cytiva, #17544602, Uppsala, Sweden) for density gradient centrifugation (400×g, 30 min). The splenic mononuclear cells at the interface between the two phases were collected, washed with DPBS, and counted on a hemocytometer.

These cells were seeded (4×10^6 cells/well) in a 6-well plate and rested for 2 h. They were then stimulated with PMA (50 ng/mL) and ionomycin (1 μM) (Solarbio, I8800, Beijing, China) over time, and the NF-κB activation and total M1-linked ubiquitination were assessed at different time points by immunoblotting.

Culture of mouse bone marrow-derived macrophages (BMDMs)

Femurs and tibias from the WT and R/K mice (8-12 weeks) were collected and cut at both ends; the marrow cavities were rinsed with cooled PBS, and the collected solution was filtered through a 70-μm mesh. After centrifugation, the cells were resuspended in IMDM medium supplemented with macrophage colony-stimulating factor (M-CSF, 20 ng/mL) and cultured in a dish in an incubator at 37 °C with 5% CO_2. On day 3, a fresh medium (equal to half volume of the culture medium) containing M-CSF was added. After 7 days, differentiated macrophages attached to the bottom of the dish were harvested, centrifuged, and counted on a hemocytometer. These BMDMs (1×10^6 cells/well) were seeded in a 12-well plate and were polarized into M1- or M2-type macrophages by adding corresponding stimuli (LPS+IFN-γ for M1, IL-4+IL-13 for M2) for subsequent experiments.

Preparation of purified recombinant C-HOIL-1 and STAT1 proteins for in vitro pull-down experiments

The DNA fragment encoding C-HOIL-1 was amplified by PCR from the full-length human HOIL-1 gene. The gene fragment was inserted into a plasmid, and the His-tagged recombinant C-HOIL-1 protein was expressed in Escherichia coli BL21 (DE3) strain. The expression was induced by isopropyl-β-d-thiogalactopyranoside (IPTG) (250 μM) (Aladdin, Shanghai, China) with the addition of $ZnCl_2$ (250 μM) (SCR, Shanghai, China) at 16 °C. The crude proteins were initially purified by Ni-NTA beads (Smart-lifesciences, Changzhou, China); the protein eluates

were then concentrated to a volume of 2 mL using a centrifugal filter unit (molecular weight cut-off (MWCO) 10,000 Da, Amicon, Merck, Darmstadt, Germany). The concentrated proteins were loaded onto a Superdex 200 Increase 10/300 GL (Cytiva, Uppsala, Sweden) column. The fractions of the first eluted main peaks were pooled to obtain the highly purified C-HOIL-1 proteins.

Similarly, the GST-tagged recombinant STAT1 proteins were expressed at 18 °C overnight with the induction of IPTG (250 μM). The bacterial lysates were purified with glutathione beads (Smart-lifesciences, Changzhou, China), and the eluted STAT1 proteins were diluted three folds in the heparin buffer A and loaded onto a 5-mL HiTrap Heparin HP column (Cytiva). Proteins were eluted with the heparin buffer B, and the collected fractions were concentrated to a volume of 2 mL using centrifugal filter units (MWCO 30,000 Da, Amicon). The proteins were further loaded onto a Superdex 75 or Superdex 200 16/600 GL size-exclusion chromatography column (Cytiva) equilibrated with the Tris-HCl buffer (20 mM) at pH 8.0 containing 200 mM NaCl and 1 mM EDTA. Purified STAT1 proteins were concentrated (MWCO 3,000 Da, Amicon) and flash-frozen in the liquid nitrogen.

The direct interaction between C-HOIL-1 and STAT1 was examined by GST pull-down assay in a buffer containing 20 mM Tris-HCl (pH 7.5) and 150 mM NaCl. GST-tagged STAT1 proteins at a final concentration of 20 μg (in a total volume of 400 μL) were immobilized and precipitated by the addition of fresh glutathione sepharose beads (30 μL) (Smart-lifesciences, Changzhou, China). The beads were washed three times with 1 mL of the same pull-down buffer, followed by incubation with 40 μg C-HOIL-1 proteins for 1 h on ice. The beads were then washed four times and boiled in 30 μL of loading buffer prior to the protein separation in SDS-PAGE. The separated proteins were detected by Coomassie Brilliant Blue (CBB) (Macklin, Shanghai, China) staining and immunoblotting with anti-His antibody (CST, Danvers, MA, USA).

2. Supplementary figures

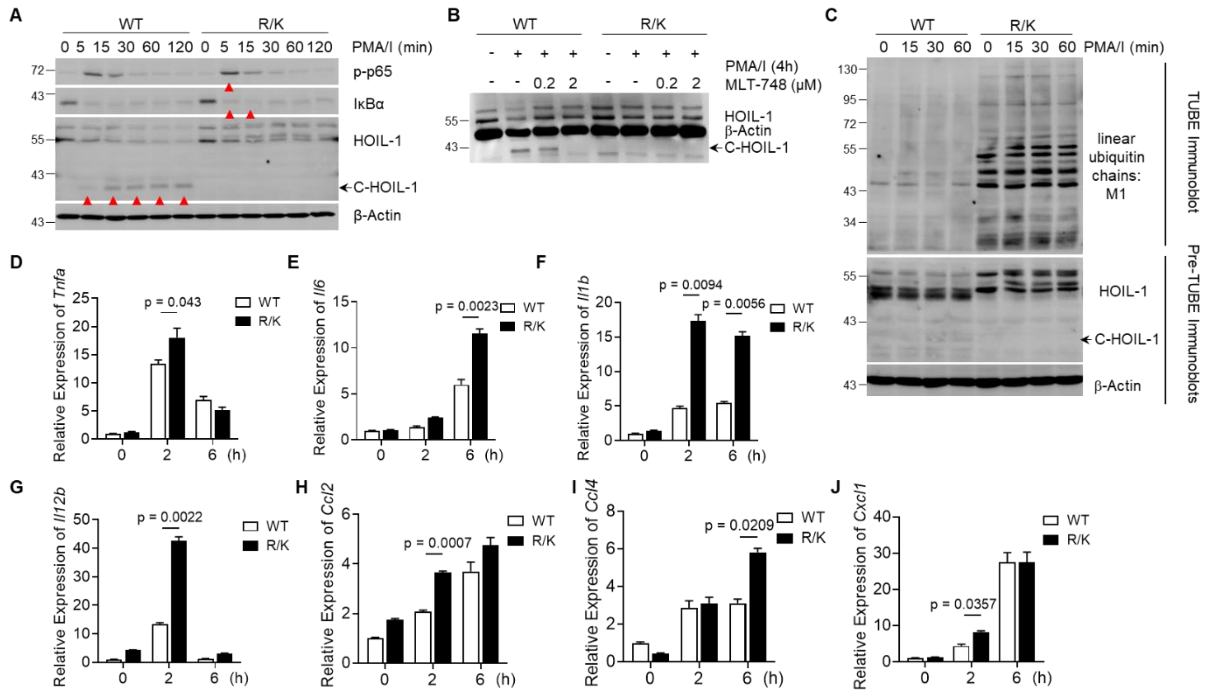

Figure S1. The abnormality of HOIL-1 cleavage promoted NF-κB activation in splenic mononuclear cells from the R/K mice. (**A**) Immunoblots showing the increase in the phosphorylation of p65 (p-65) and the degradation of IκBα (indicated by red arrows) for NF-κB activation in splenic mononuclear cells from the R/K mice upon PMA and ionomycin (PMA/I) stimulation over time compared with those from the WT mice; the cleaved C-HOIL-1 fragment was observed in the WT group, but not in the R/K group; β-Actin as the internal control. (**B**) Immunoblots showing the decreased C-HOIL-1 band by the MALT1 inhibitor MLT-748 pretreatment (2 μM) under PMA/I stimulation in splenic mononuclear cells from the WT mice, while no C-HOIL-1 bands were observed in those from the R/K mice; β-Actin as the internal control. (**C**) Immunoblots showing up-regulated linear (M1-linked) polyubiquitin chains enriched by TUBE magnetic beads in splenic mononuclear cells from the R/K mice than those from the WT mice upon PMA/I stimulation; β-Actin as the internal control. (**D-J**) The relative mRNA expressions of *Tnfa* (**D**), *Il6* (**E**), *Il1b* (**F**), *Il12b* (**G**), *Ccl2* (**H**), *Ccl4* (**I**) and *Cxcl1* (**J**) were increased in the R/K splenic mononuclear cells when compared with WT ones upon PMA/I stimulation for 0, 2 and 6 h by RT-qPCR. N = 3; PMA = 50 ng/mL, ionomycin = 1 μM.

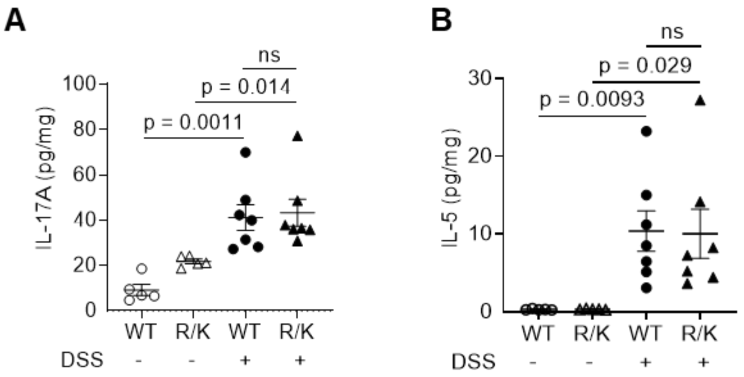

Figure S2. Production of IL-17A and IL-5 cytokines in the WT and R/K mice under DSS-induced colitis. Levels of the cytokines IL-17A (**A**) and IL-5 (**B**) in the colon tissues of WT and R/K mice with or without DSS-induced colitis (Day 9) by ELISA. N = 5 for water groups, N = 7 for DSS groups; ns: not significant.

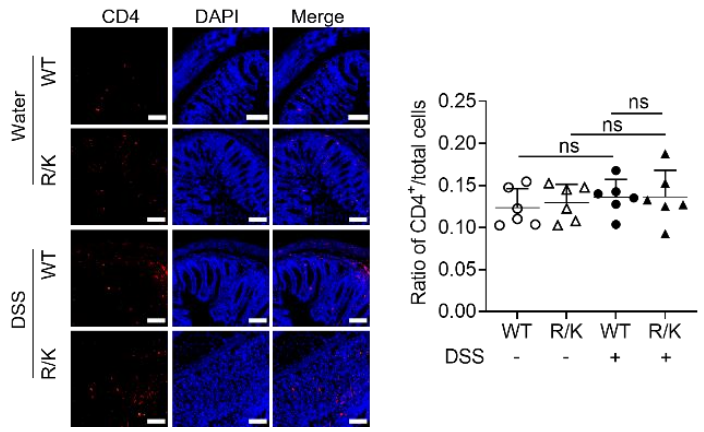

Figure S3. The infiltration of CD4^{+}T cells in the colon of WT and R/K mice under DSS-induced colitis. Immunofluorescence images of the colon sections (left) showing the infiltration of CD4^{+} T cells (red) to the colon of WT and RK mice under DSS-induced colitis; the ratio of CD4^{+} T cells to the total cells was quantified (right); the cell nucleus was stained in blue by DAPI. Scale bar = 100 μm; N = 6 per group; ns: not significant.

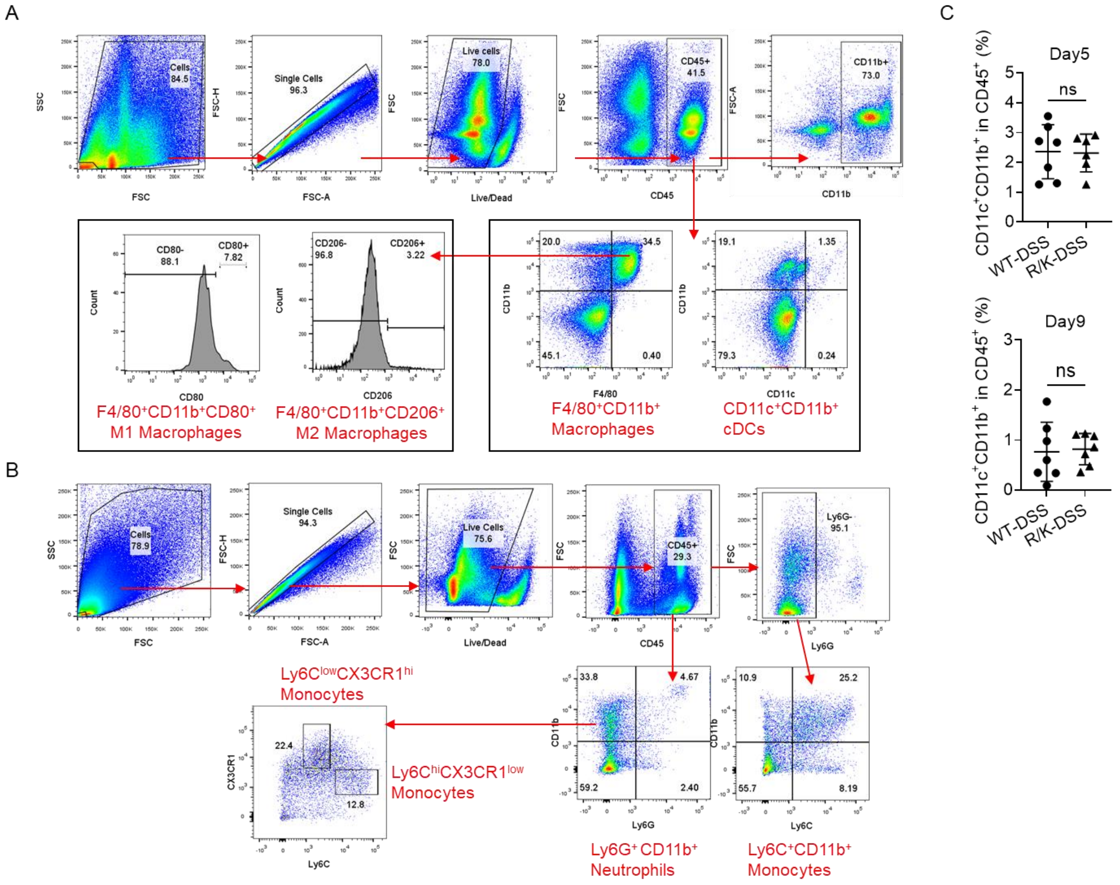

Figure S4. The multicolor flow analysis identifying different myeloid cell populations in the colonic lamina propria. (**A, B**) The gating strategy for identifying macrophages (F4/80$^+$CD11b$^+$) and their M1 (CD80$^+$)/M2 (CD206$^+$) sub-types as well as the classical dendritic cells (cDCs, CD11c$^+$CD11b$^+$) (**A**), and monocytes (Ly6C$^+$CD11b$^+$) and their sub-types (CX3CR1^{low}Ly6C^{hi}, CX3CR1^{hi}Ly6^{low}) and neutrophils (Ly6G$^+$CD11b$^+$) (**B**) in the colonic lamina propria of the WT and RK mice. (**C**) Flow cytometry analysis on the differences in the percentage of cDCs in the colonic lamina propria between the WT-DSS and R/K-DSS groups on Day 5 (top) and Day 9 (bottom) after DSS induction; N = 7. ns: not significant.

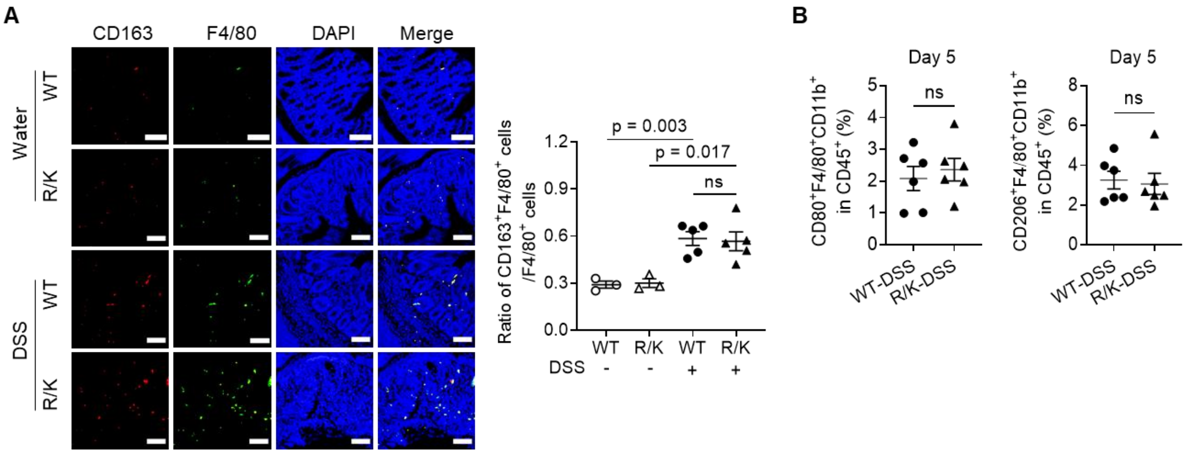

Figure S5. The infiltration of different sub-types of macrophages in the colon. (**A**) Immunofluorescence images showing the ratio of $CD163^+F4/80^+$ M2-type macrophages in the colon tissue of the WT and R/K mice with or without DSS-induced colitis; CD163 and F4/80 were stained in red and green, respectively, while the cell nucleus was stained in blue (by DAPI); the quantitative analysis was shown on the right; scale bar = 100 μm; N = 3 for water groups, N = 5 for DSS groups. (**B**) The differences in the percentage of $CD80^+F4/80^+CD11b^+$ M1-type macrophages (left) and $CD206^+F4/80^+CD11b^+$ M2-type macrophages (right) in the colonic lamina propria between the WT-DSS and R/K-DSS group on Day 5 by the flow cytometry analysis; N = 6. ns: not significant.

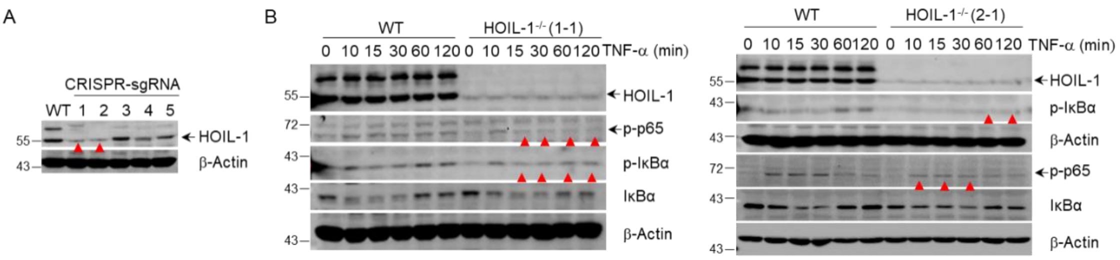

Figure S6. Construction and validation of HOIL-1$^{-/-}$ THP-1 cells by CRISPR/Cas9 approach. (**A**) Immunoblots showing the deletion of HOIL-1 by different sgRNAs in THP-1 cells; red arrows indicated the decrease in HOIL-1 expression; β-Actin as the internal control. (**B**) Immunoblots showing the decrease (indicated by red arrows) in the phosphorylation of p65 (p-65) and IκBα (p-IκBα) and the degradation of IκBα of NF-κB activation in HOIL-1$^{-/-}$ (1-1 and 2-1 clones) cells compared with WT cells upon TNF-α (20 ng/mL) stimulation over time (0-120 min); β-Actin as the internal control.

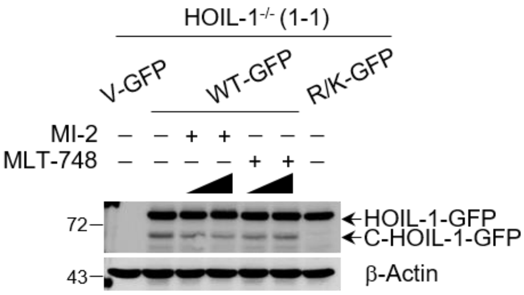

Figure S7. MALT1-dependent cleavage of HOIL-1 in THP-1 cells. Immunoblots showing that pretreatment (30 min) of MALT1 inhibitors MI-2 (10 and 20 μM) and MLT-748 (2 and 5 μM) reduced the cleaved C-HOIL-1-GFP in HOIL-1$^{-/-}$(1-1) cells expressing WT-HOIL-1 (WT-GFP), but no C-HOIL-1-GFP was observed in the cells expressing R/K-HOIL-1 (R/K-GFP); cells transduced with empty lentiviral particles (V-GFP) as the negative control; β-Actin as the internal control.

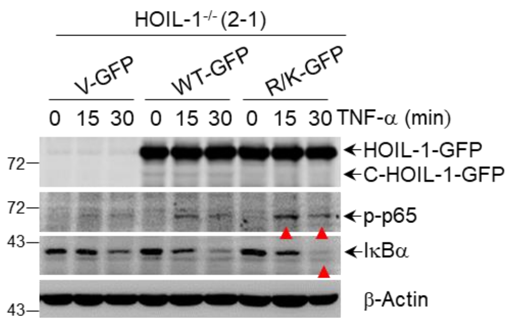

Figure S8. Negative regulation of NF-κB signaling by HOIL-1 cleavage. The immunoblots showing the increased phosphorylation of p65 (p-p65) and the degradation of IκBα for NF-κB activation upon TNF-α (20 ng/mL) stimulation in HOIL-1$^{-/-}$ (2-1) cells expressing R/K-HOIL-1 (R/K-GFP) when compared with WT-HOIL-1 (WT-GFP) by immunoblotting; cells transduced with empty lentiviral particles (V-GFP) as the negative control; β-Actin as the internal control.

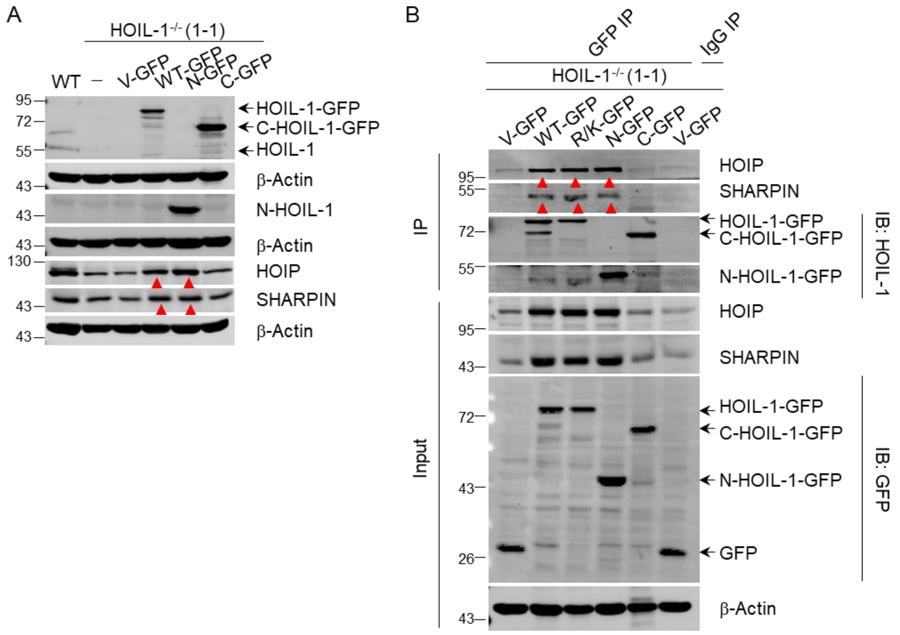

Figure S9. The inability of C-HOIL-1 in interacting and stabilizing HOIP and SHARPIN proteins. (**A**) Immunoblots showing the restored levels (indicated by red arrows) of HOIP and SHARPIN in HOIL-1$^{-/-}$(1-1) cells by the full-length WT-HOIL-1 (WT-GFP) and N-HOIL-1 (1-165 aa) (N-GFP), but not by C-HOIL-1 (165-510 aa) (C-GFP); cells transduced with the empty vector (V-GFP) as the control; β-Actin as the internal control. (**B**) Immunoblots demonstrating the pulled-down HOIP and SHARPIN by the full-length WT-HOIL-1 (WT-GFP) and R/K HOIL-1 (R/K-GFP) as well as N-HOIL-1 (N-GFP) through co-immunoprecipitation of GFP,

but not by C-HOIL-1 (C-GFP); cells expressing empty vector (V-GFP) enriched by GFP or IgG antibodies were used as the controls; β-Actin as the internal control.

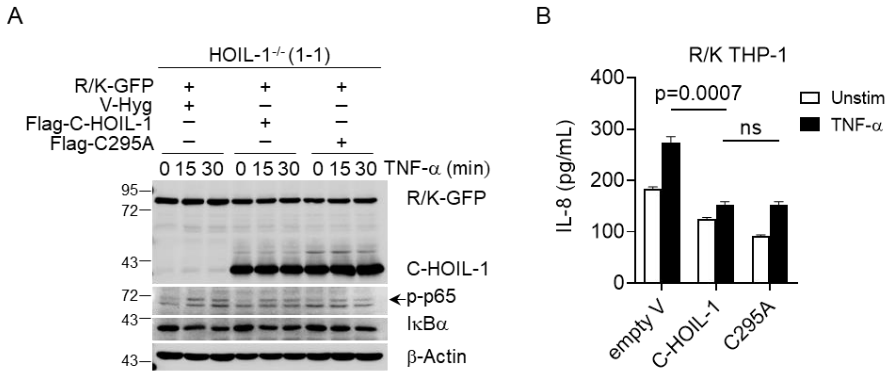

Figure S10. The effects of mutant C-HOIL-1 on NF-κB activation and IL-8 production. (**A**) Immunoblots showing NF-κB activation (p-p65 and IκBα degradation) in R/K-GFP cells expressing V-Hyg, Flag-C-HOIL-1 or Flag-C295A (enzymatically inactive C-HOIL-1) upon TNF-α (20 ng/mL) stimulation over time; β-Actin as the internal control. (**B**) The ELISA measurements on the production of IL-8 in R/K-GFP cells expressing V-Hyg, Flag-C-HOIL-1 or Flag-C295A with/without TNF-α (20 ng/mL) stimulation for 24 h; N = 3. ns: not significant.

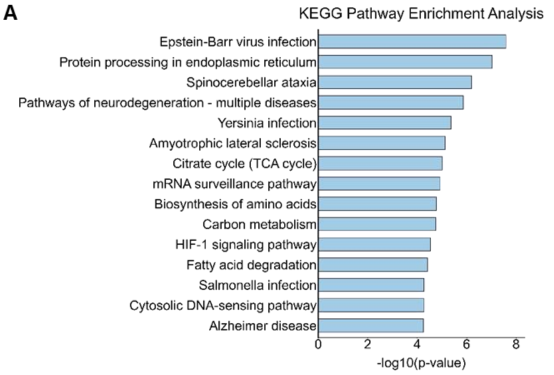

Description	p.adjust	Count	GeneSymbols
Epstein-Barr virus infection	8.49E-06	31	STAT1;PSMC2;PSMC3;PSMD11;PSMD6;PSMC4;PSMC5;TAP2;USP7;PSMD12;HLA-A; TAPBP; PSMC6; IKBKG; PSMD2;HLA-B; STAT3; PLCG2; IKBKB; PIK3R1;HLA-C; ISG15; TBK1; SIN3A; BTK; JAK1; MAVS;ADRM1; CHUK; NFKB1; IKBKE

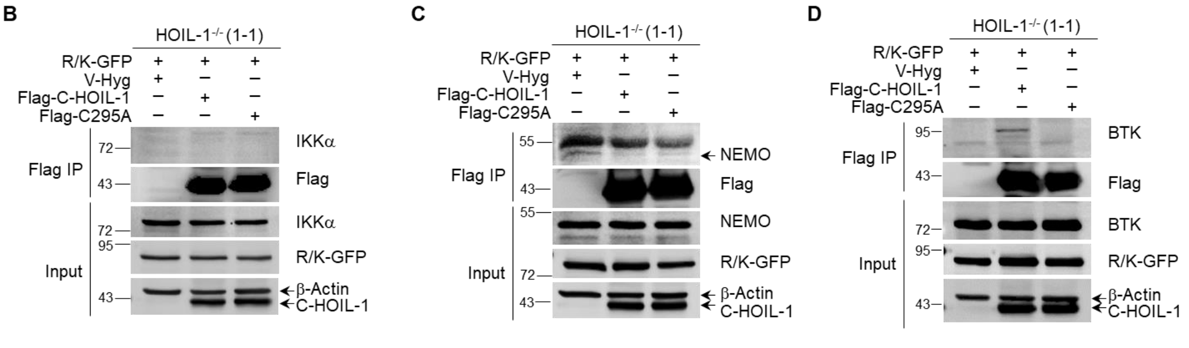

Figure S11. Validation of the C-HOIL-1 interacting proteins identified by IP-MS assay.

(**A**) The bar graph (left) showing the top 15 pathways of enriched proteins by the KEGG pathway analysis on the mass spectrometry data; detailed information for the top-ranked pathway of Epstein-Barr virus infection was listed in a table on the right. (**B-D**) The immunoprecipitation of Flag-labeled proteins to pull down IKKα (**B**), NEMO (**C**), and BTK (**D**) in R/K-GFP cells expressing V-Hyg (control), Flag-C-HOIL-1 or mutant C-HOIL-1 (Flag-C295A); β-Actin as the internal control.

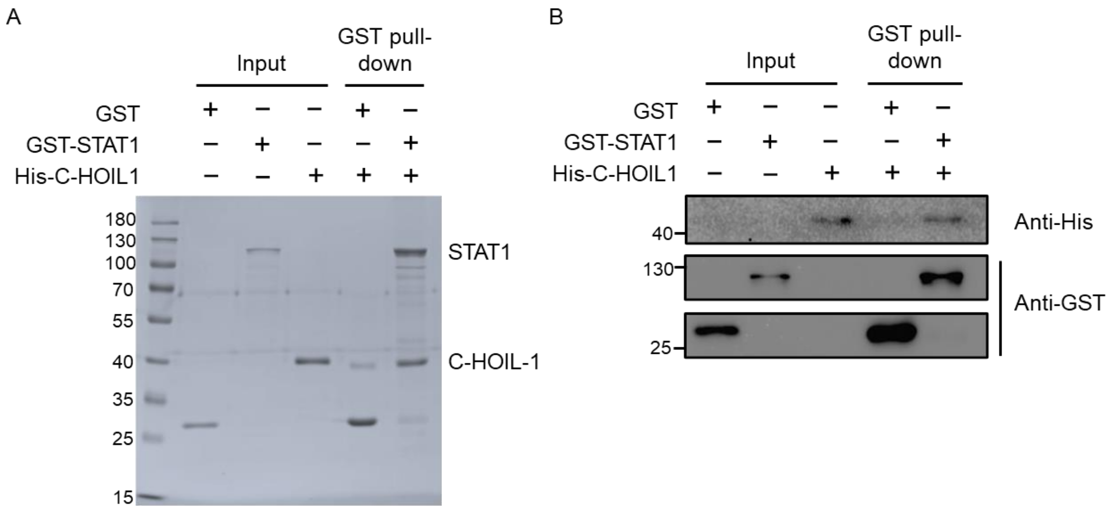

Figure S12. The direct physical interaction between C-HOIL-1 and STAT1. (**A, B**) The binding complexes captured by GST affinity resin were analyzed by Coomassie Brilliant Blue (CBB) staining (**A**) and immunoblotting (**B**) with anti-His antibody on the recombinant GST-STAT1 (bait) and His-C-HOIL1 (prey) in a test tube; GST empty vector served as the negative control.

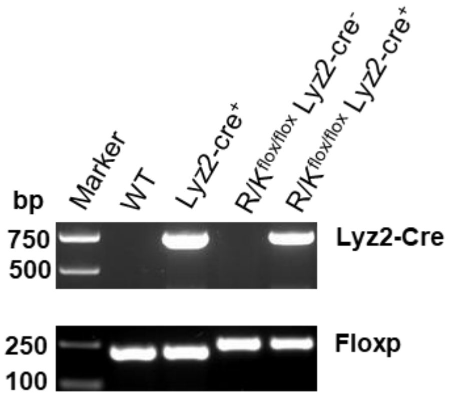

Figure S13. Genotype identification of mice with myeloid cell-specific R/K mutation. The genotype of newborn transgenic mice from breeding pairs was identified as follows: Lyz2-Cre^{+}, R/K$^{flox/flox}$Lyz2-Cre^{-} (control mice), and R/K$^{flox/flox}$Lyz2-Cre^{+} (target mice). DNA was extracted by toe clipping in mice between 10 and 21 days after birth.

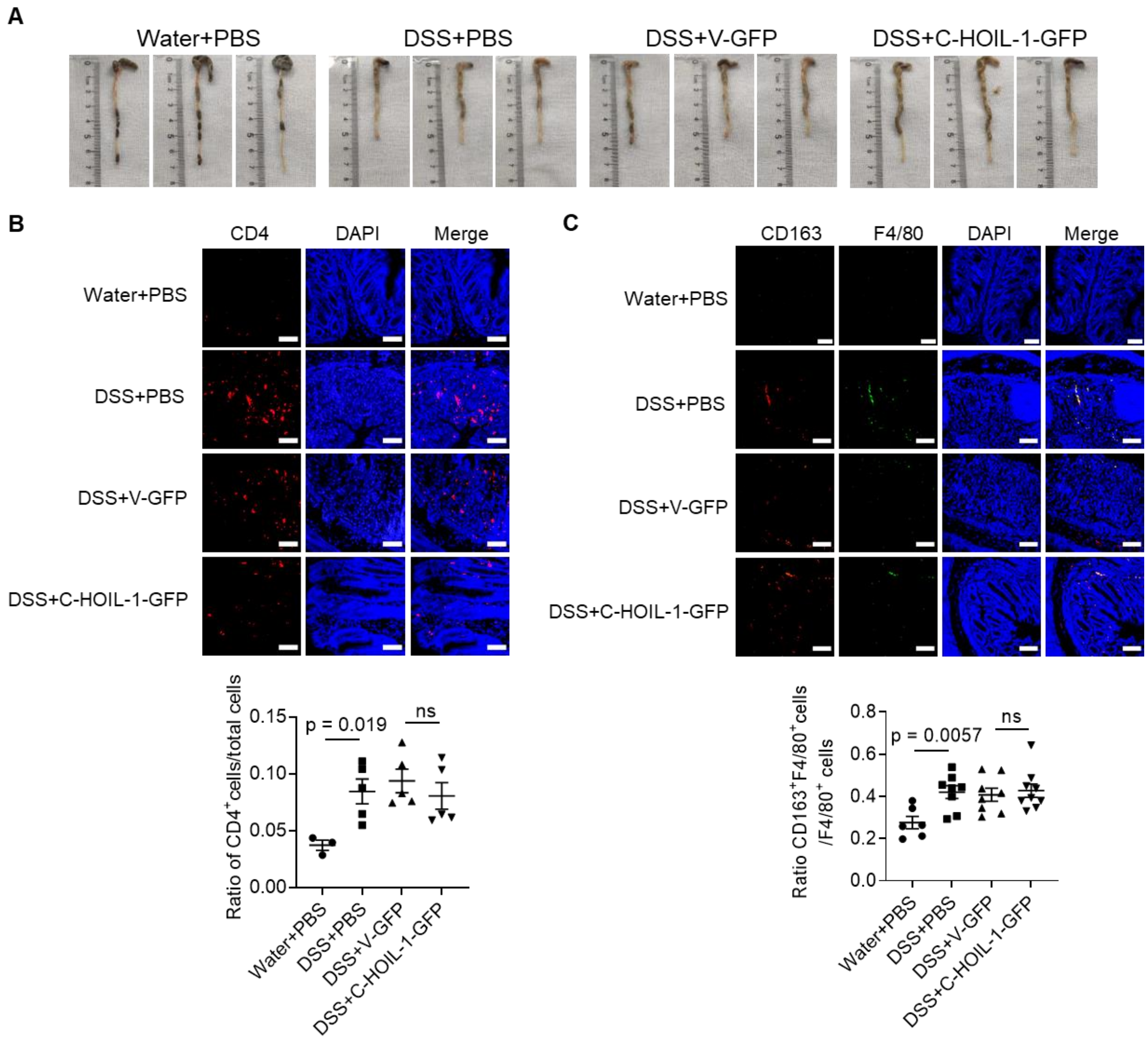

Figure S14. Effects of the pretreatment of C-HOIL-1 lentiviral particles on colon length and immune cell infiltration in the WT mice under DSS-induced colitis. (**A**) Representative photographs showing the colon length of the control mice and colitis mice with the pretreatment of C-HOIL-1-GFP or V-GFP. (**B**) Immunofluorescence images showing the percentage of $CD4^+$ T cells in the colon tissue of mice in each group: Water+PBS, DSS+PBS, DSS+V-GFP, DSS+C-HOIL1-GFP; the $CD4^+$ T cells were stained in red while the cell nucleus was stained in blue (by DAPI); the quantitative analysis was shown on the right; scale bar = 100 μm, N = 3 for water+PBS group, N = 5 for other groups. (**C**) Immunofluorescence images showing the ratio of $CD163^+F4/80^+$ M2-type macrophages in the colon tissue of mice from each group with quantitative analysis on the right; the CD163 and F4/80 were stained in red and green, respectively, while the cell nucleus was stained in blue (by DAPI); scale bar = 100 μm; N = 6 for water-PBS group, N = 8 for other groups. ns: not significant.

3. Supplementary tables

Supplementary Table S1: List of primer sequences used in this study

Gene	Forward（5'-3'）	Reverse（5'-3'）
Mouse *Rbck1*	CAAATGTACTCCTGGACTATGGCTC	TTGTTGATGAAAGTGCAGCCAG
Mouse *Rbck1*-LSL-p.R165K	CCTCTCTCTGGTTGACATTGACACAAG	AGCTCCCAAACACGGACTCCA
Mouse Lyz2-Cre	CCCAGAAATGCCAGATTACGCAC	CTTGGGCTGCCAGAATTTCTC
pLVX-IRES-Flag-C-HOIL-1-Mut-Hyg (C295A)	GAAGGACGGCGCAGACTGGATCC	TTCTGTACCACGATCTGG
Mouse *Gapdh*	CATCACTGCCACCCAGAAGACTG	ATGCCAGTGAGCTTCCCGTTCAG
Mouse *Tnfa*	CAGGCGGTGCCTATGTCTC	CGATCACCCCGAAGTTCAGTAG
Mouse *Il6*	CTGCAAGAGACTTCCATCCAG	AGTGGTATAGACAGGTCTGTTGG
Mouse *Il1b*	GAAATGCCACCTTTTGACAGTG	TGGATGCTCTCATCAGGACAG
Mouse *Il12b*	CCTGTGACACGCCTGAAGAAGATG	CTTGTGGAGCAGCAGATGTGAGTG
Mouse *Ccl2*	TTAACGCCCCACTCACCTGCTG	GCTTCTTTGGGACACCTGCTGC
Mouse *Ccl4*	TCTGTGCAAACCTAACCCCG	GAGGGTCAGAGCCCATTGGT
Mouse *Cxcl1/Kc*	TCCAGAGCTTGAAGGTGTTGCC	AACCAAGGGAGCTTCAGGGTCA
Human *TNFA*	CCTCTCTCTAATCAGCCCTCTG	GAGGACCTGGGAGTAGATGAG
Human *NOS2*	TTCAGTATCACAACCTCAGCAAG	TGGACCTGCAAGTTAAAATCCC
Human *GAPDH*	GTCTCCTCTGACTTCAACAGCG	ACCACCCTGTTGCTGTAGCCAA
Human *GBP2*	GTTCCTACATCCTCAGCCATTCC	CCACTGCTGATGGCATTGACGT
Human *CXCL9*	CCAGTAGTGAGAAAGGGTCGC	AGGGCTTGGGGCAAATTGTT
Human *CXCL10*	GGTGAGAAGAGATGTCTGAATCC	GTCCATCCTTGGAAGCACTGCA
Human *IRF1*	GCAGCTACACAGTTCCAGG	GTCCTCAGGTAATTTCCCTTCCT
Human *ARG1*	TCATCTGGGTGGATGCTCACAC	GAGAATCCTGGCACATCGGGAA
Human *IL10*	GACTTTAAGGGTTACCTGGGTTG	TCACATGCGCCTTGATGTCTG

Supplementary Table S2: Criteria for DAI scoring

Score	0	1	2	3	4
Weight loss	< 1%	1-3%	3-6%	6-9%	> 9%
Fecal morphology	Normal	Loose	Very loose	Diarrhea	Solid free
Rectal bleeding	No blood in stool	Blood can be detected by blood test strip in stool	Observed blood in stool	A lot of blood in stool	Blood observed in stool and around the anus

Supplementary Table S3: Criteria for the histological assessment and scoring

Grade	Loss of goblet cells	Submucosal edema	Crypt abscesses	Extend of crypt damage	Infiltration of inflammatory cells	Extend of inflammation	Reactive epithelial hyperplasia
0	None	None	None	None	None	None	None
1	Focal (≤ 3)	Focal (≤ 3)	Focal (≤ 3)	Basal one third	Focal (≤ 3)	Mucosa	Focal (≤ 3)
2	Multifocal (4-10)	Multifocal (4-10)	Multifocal (4-10)	Basal two thirds	Multifocal (4-10)	Mucosa + submucosa	Multifocal (4-10)
3	Diffuse (> 10)	Diffuse (> 10)		Entire crypt damage	Diffuse (> 10)	Mucosa + submucosa + muscle layer	Diffuse (> 10)
4				Crypt damage + ulceration		Transmural	

Supplementary Table S4: List of antibodies information in this study

Antibodies used for immunoblotting		Source	Cat Number
β-Actin		CST	#8457; #3700
GAPDH		CST	#2118; #97166
HOIL-1		Millipore	MABC576
HOIL-1		Sigma	HPA024185
Phospho-p65		CST	#3031; #3033
IκBα		CST	#9242
Phospho-IκBα		CST	#9246
RNF31/HOIP		Abcam	ab46322
SHARPIN		CST	#12541
GFP		CST	#2956
FLAG		CST	#14793
Linear Polyubiquitin		Lifesensor	AB130
K48-linkage Specific Polyubiquitin		CST	#8081
STAT1		CST	#14994
Phospho-STAT1		CST	#9167
IKKα		CST	#61294
IKKγ/NEMO		CST	#2685
IKKε		CST	#2905
IRDye® 680RD Goat anti-Mouse IgG		LI-COR	#926-68070
Anti-Rabbit IgG (GOAT) Antibody DyLight 800		Rockland	#611-145-002
Caspase-1 (H)		CST	#3866
Cleaved-Caspase-1 (M)		CST	#89332
ARG1		CST	#93668
**Antibodies used for immunofluorescence**		**Source**	**Cat Number**
F4/80 Monoclonal Antibody (SP115)		Thermo	MA5-16363
F4/80 Monoclonal Antibody (Cl:A3-1)		Thermo	MA5-16630
CD11b Recombinant Rabbit Monoclonal Antibody		Abcam	Ab133357
Anti-Myeloperoxidase, MPO		Abcam	ab208670
CD4 Polyclonal Antibody		Thermo	PA5-85858
FLAG		CST	#8146
Goat anti-Rabbit IgG (H+L) Cross-Adsorbed Secondary Antibody, Alexa Fluor™ 568		Thermo	A-11011
Goat anti-Rat IgG (H+L) Highly Cross-Adsorbed Secondary Antibody, Alexa Fluor™ 488		Abcam	ab150165
Goat anti-Mouse IgG (H+L) Highly Cross-Adsorbed Secondary Antibody Alexa Fluor™ 568		Thermo	A-11004
Donkey anti-Rabbit IgG H&L Alexa Fluor® 647		Abcam	ab150075
**Antibodies used for flow cytometry**	**Clone**	**Source**	**Cat Number**
BV650 Rat Anti-Mouse CD45	30-F11	BD	563410
PE/Cyanine7 anti-mouse/human CD11b	M1/70	Biolegend	101215
PE anti-mouse F4/80	BM8	Biolegend	123110
FITC Rat anti-Mouse Ly-6G	1A8	BD	551460

BV421 Hamster Anti-Mouse CD11c	HL3	BD	562782
Brilliant Violet 510™ anti-mouse Ly-6C	HK1.4	Biolegend	128033
anti-mouse CX3CR1-FITC	SA011F11	Biolegend	149020
Alexa Fluor® 647 anti-mouse CD206	C068C2	Biolegend	141712
Brilliant Violet 510™ anti-mouse CD80	16-10A1	Biolegend	104741
LIVE/DEAD™ Fixable Blue Dead Cell Stain Kit		Thermo	L34962
TruStain FcX™ (anti-mouse CD16/32) Antibody		Biolegend	1013220

Supplementary Table S5: SgRNA sequences

Term	Sequence（5‘-3’）
sgRNA -1	AGAGACGCCACTGTCATATCAGG
sgRNA -2	AGTGCGCCCTGATATGACAGTGG
sgRNA -3	ATCAACAAGCCCACGCGGCCTGG
sgRNA -4	CGCCTCATACCAGCCCGACGAGG
sgRNA -5	CGTCGGGCTGGTATGAGGCGGGG
